# Supplementary material for: Changes in ocean health in British Columbia from 2001 to 2016
Source: PLoS One. 2020 Jan 30;15(1):e0227502. doi: 10.1371/journal.pone.0227502 (PMC6992189; doi:10.1371/journal.pone.0227502)
Supplement: S1 File — (PDF) [file pone.0227502.s001.pdf]

Supporting Information: Changes in ocean health in British Columbia from 2001 to 2016

Supporting Figures

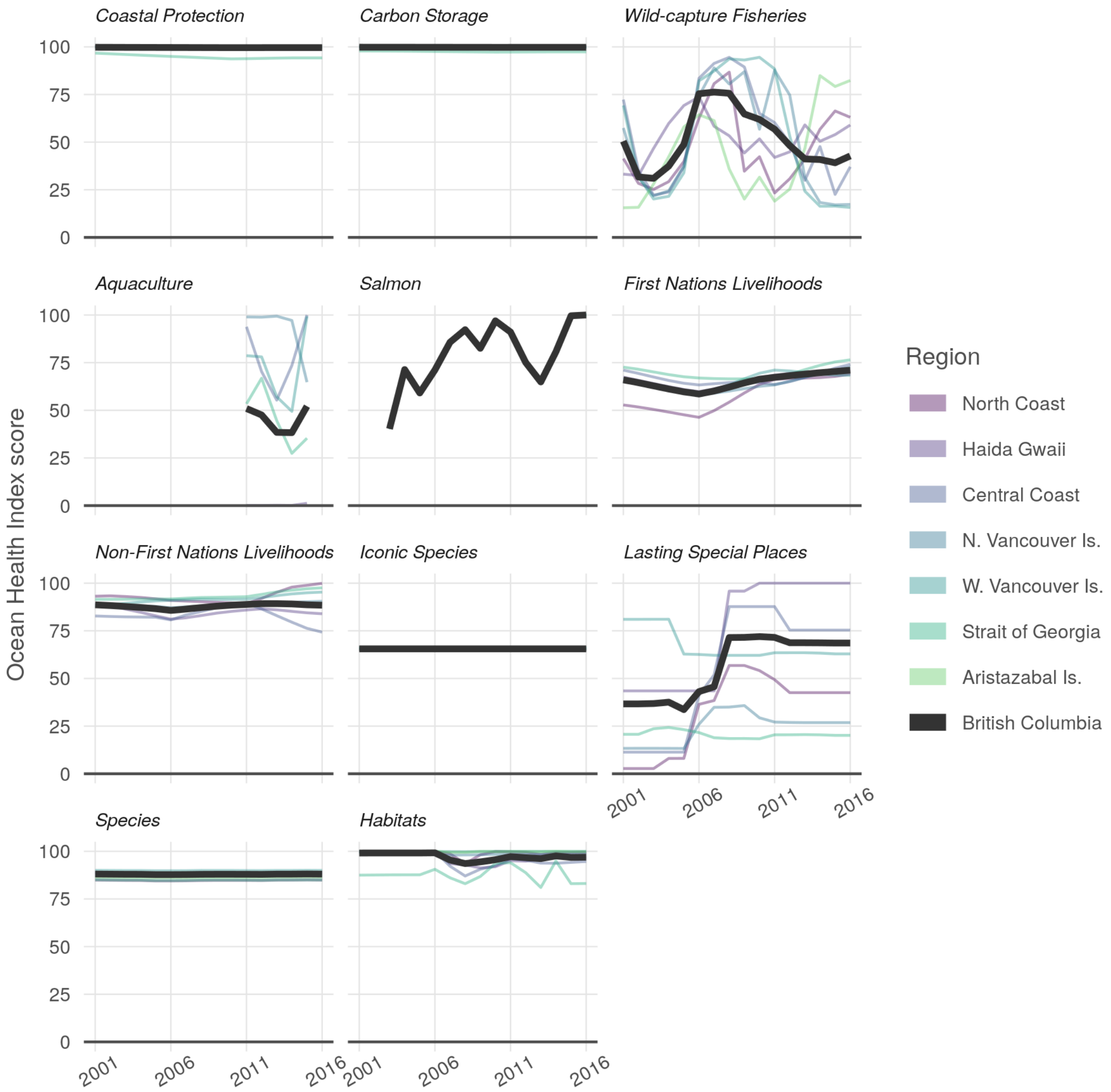

S1 Fig.: Subgoal scores over time (goal scores are in the main manuscript, Fig. 4). The heavy dark line indicates BC-level scores for each subgoal; the thinner lines represent region-level scores for each subgoal.

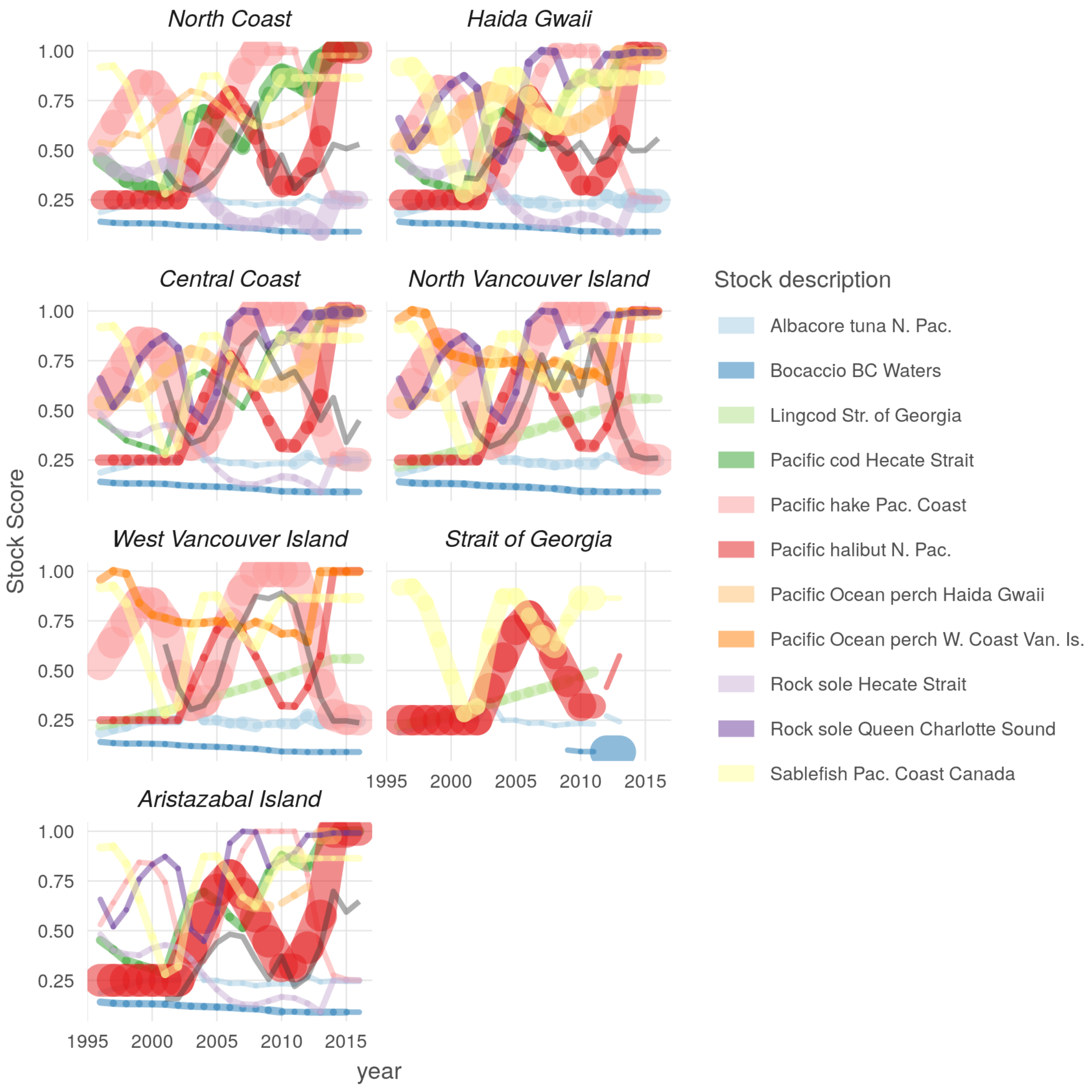

S2 Fig.: Wild-capture fisheries stock scores over time. Black line indicates overall catch-weighted score for the Wild-Capture Fisheries goal per region. Line thickness represents relative catch of each stock over time. Note that while some assessed stocks are present in the Strait of Georgia region, unassessed stocks dominate (> 90% of overall catch in the region) and so the region is not assigned a score for this goal.

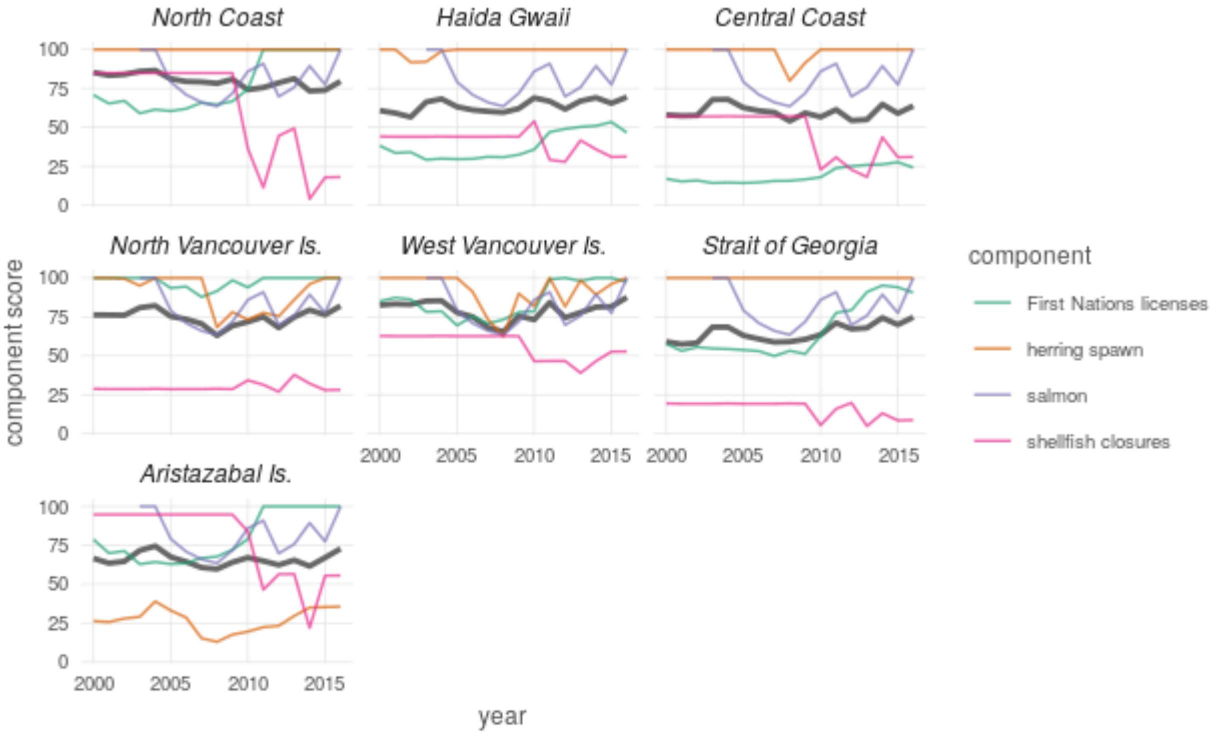

S3 Fig.: Component scores for First Nations Resource Access Opportunity goal. The heavy grey line indicates the overall status calculated as the unweighted average of all components.

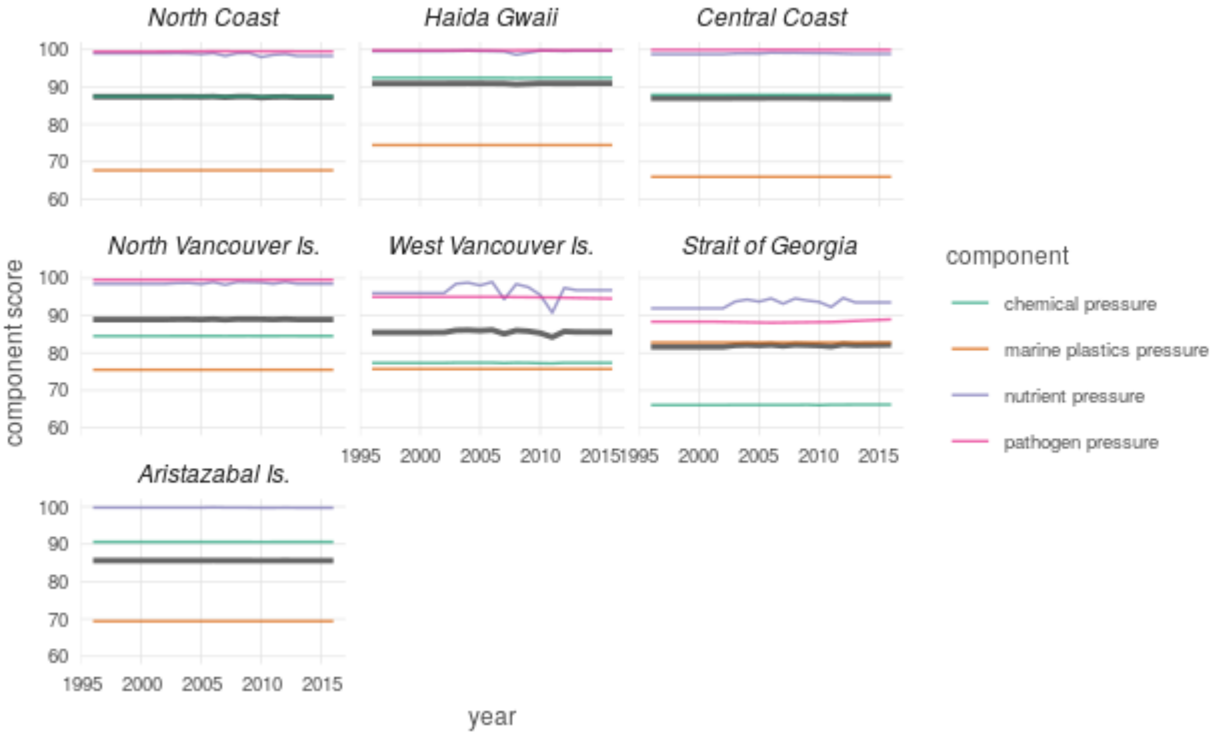

S4 Fig.: Components used to calculate Clean Waters score. The heavy grey line represents the goal status, calculated as the geometric mean of the component scores. Note the vertical scale begins at a score of 60.

Supporting Tables

S1 Table: Status layers

targets	name	units	description	ref
Coastal Protection	Exposure-weighted coastal forest extent	proportion	Current extent of coastal forest habitat relative to historical extent weighted by exposure	[S1-3]
	Exposure-weighted saltmarsh extent	proportion	Current extent of saltmarsh habitat relative to historical extent weighted by exposure	[S1-3]
Carbon Storage	Coastal forest extent	proportion	Current extent of coastal forest habitat relative to historical extent	[S1,4]
	Saltmarsh extent	proportion	Current extent of saltmarsh habitat relative to historical extent	[S1,4]
Wild-Capture Fisheries	B/Bmsy estimates (from RAM)	ratio	The ratio of fish population abundance compared to the abundance required to deliver maximum sustainable yield	[S5]
	Catch estimates (from DFO)	tonnes	Estimate of total annual catch for a given fishery	[S6]
	F/Fmsy estimates (from RAM)	ratio	The ratio of fishery harvest relative to the fishery harvest at maximum sustainable yield	[S5]
	Proportional area of stock by region	proportion	Spatialized stock area for weighting RAM-reported catch across OHIBC regions	[S7]
Aquaculture	Mariculture harvest	tonnes	Tonnes of mariculture harvest	[S8]
	Aquaculture production potential	t/sq km/year	Aquaculture production potential for finfish and shellfish at different reference levels	[S9]
	Aquaculture tenure area	sq km	DFO aquaculture tenures	[S10]
Wild-Capture Salmon	Salmon catch/catch target	ratio	Salmon catch relative to catch target as proxy for maximizing sustainable yield for commercial fishing purposes	[S11-13]
First Nations Resource Access Opportunities	Shellfish closure days	days	Shellfish closure days area-weighted by region as a proxy for access to shellfish gardens	[S14]
	FN Commercial fishing licenses	proportion	Commercial fishing licenses allocated to First Nations (as proportion of total licenses) as a measure of access to commercial fisheries	[S15]
	FN Commercial fishing licenses	proportion	Commercial fishing licenses allocated to First Nations (proportion relative to FN proportion of population) as a measure of access to commercial fisheries	[S15]
	Salmon escapements/escape target	ratio	Salmon escapements relative to escapements target as a proxy for sustainable biomass for First Nations FSC access	[S11-13]
	Herring Spawn Habitat Index	abundance per km	Herring spawn habitat index as a proxy for opportunity to collect herring spawn	[S16]
First Nations Livelihoods	Median household income (First Nations)	2016 Can$	Population-weighted mean of CPI-adjusted median household income for First Nations communities	[S17]
	Unemployment rate (First Nations)	proportion	Mean unemployment rate for First Nations communities	[S17]
Non-First Nations Livelihoods	Median household income (non-FN)	2016 Can$	Population-weighted mean of CPI-adjusted median household income for non-FN communities	[S17]
	Unemployment rate (non-FN)	proportion	Mean unemployment rate for non-FN communities	[S17]

S1 Table cont'd: Status layers

targets	name	units	description	ref
Tourism & Recreation	Park visitors	count	Number of visitors to provincial parks in OHIBC regions	[S18]
	Visitor center visitors	count	Number of visitors to visitor centers in OHIBC regions	[S19]
Iconic Species	Iconic Species by region	presence absence	List of iconic species and the regions in which they are found according to IUCN or Aquamaps	[S20-22]
	IUCN or BCSEE extinction risk score	scaled 0-1	IUCN or BCSEE scored extinction risk category for iconic species per assessment year	[S20,23]
	IUCN and COSEWIC trend	text or numeric	IUCN text field of population trend OR COSEWIC species health time series trend	[S20,23]
Lasting Special Places	Inland coastal protected areas	sq km	Protected areas located 1 km inland	[S24]
	Offshore coastal protected areas	sq km	Protected areas located within 3 nmi offshore	[S24]
	Total inland watershed area	sq km	Inland area of OHI regions within coastal subwatersheds	[S25]
	Total offshore 3 nmi area	sq km	Offshore area of OHI regions within 3 nmi of shoreline	[S26]
Species	Species population trend	stable incr decr	Species population trend based on most recent IUCN Red List assessment	[S20,23]
	Species range as pct of region	ratio	Species range as a proportion of region area as determined by IUCN or AquaMaps range maps	[S20-22]
	Species risk category score	scaled 0-1	Species risk score based on IUCN Red List extinction risk category	[S20,23]
Habitats	EBSA habitat condition	trawled area	Current condition of EBSA habitat based on trawled area	[S6,27]
	Soft Bottom habitat condition	ratio	Current condition of soft bottom habitat based on trawl effort	[S2,6]
	Saltmarsh habitat condition	ratio	Current condition of saltmarsh habitat relative to historical condition	[S1]
Clean Waters	Coastal chemical pollution	scaled 0-1	Modeled chemical pollution from commercial shipping traffic, ports and harbors, land-based pesticide use (organic pollution), and urban runoff (inorganic pollution)	[S28]
	Coastal nutrient pollution	scaled 0-1	Modeled data based on fertilizer consumption from the Food and Agricultural Organization	[S28]
	Pathogen pollution	scaled 0-1	population density without access to improved sanitation	[S29-32]
	Marine plastics	scaled 0-1	Global marine plastics	[S33]
	Region areas based on EEZ boundaries	sq km	Area of OHIBC regions modified from MaPP boundaries within BC exclusive economic zone	[S26]
	Regions	region name	Region names by region ID	[S26]

S2 Table: Pressures layers

name	units	description	ref
Aquaculture benthic pressures	scaled 0-1	Rescaled harvest-weighted benthic impacts due to aquaculture	[S34]
Aquaculture incidental harvest	scaled 0-1	Rescaled harvest-weighted incidental take of fish species due to aquaculture	[S34]
Aquaculture mammal take	scaled 0-1	Rescaled harvest-weighted number of mammals drowned or taken as predator control due to aquaculture	[S34]
Ocean acidification	scaled 0-1	Ocean acidification pressure scaled using biological thresholds	[S28]
Sea level rise	scaled 0-1	Sea level rise pressure	[S28]
Sea surface temperature anomalies	scaled 0-1	Sea surface temperature anomalies	[S28]
UV radiation pressure	scaled 0-1	Modeled UV radiation based on Erythemal UV Irradiance data provided by GES DISC.	[S28]
FN Res. Access Opp. component weight	scaled 0-1	Contribution of each component to the First Nations Resource Access Opportunities score	[S26]
Coastal protection weights	scaled 0-1	Habitat extent multiplied by habitat protection rank for coastal forests and saltmarsh	[S26]
Carbon storage weights	scaled 0-1	Habitat extent multiplied by carbon storage capacity for coastal forests and saltmarsh	[S26]
Habitat presence	boolean	List of habitats in each region	[S26]
Discards	scaled 0-1	Pressure that non-targeted catch exerts on the system	[S35]
Landings	scaled 0-1	Pressure that targeted catch exerts on the system	[S35]
Intertidal habitat destruction	scaled 0-1	Coastal population density (25 mi from shore) as a proxy for intertidal habitat destruction	[S17]
Subtidal softbottom habitat destruction	scaled 0-1	Demersal destructive commercial fishing practices (i.e., trawling) in softbottom habitat as a proxy for soft bottom habitat destruction	[S2,6]
Logging intensity in OHIBC watersheds	scaled 0-1	Logging impact per area compared to the overall inland region area	[S36]
Chemical pollution	scaled 0-1	Modeled chemical pollution from commercial shipping traffic, ports and harbors, and pesticide use	[S28]
Coastal chemical pollution	scaled 0-1	Modeled chemical pollution from commercial shipping traffic, ports and harbors, land-based pesticide use (organic pollution), and urban runoff (inorganic pollution)	[S28]
Nutrient pollution	scaled 0-1	Modeled data based on fertilizer consumption from the Food and Agricultural Organization	[S28]
Coastal nutrient pollution	scaled 0-1	Modeled data based on fertilizer consumption from the Food and Agricultural Organization	[S28]
Pathogen pollution	scaled 0-1	population density without access to improved sanitation	[S29-32]
Marine plastics	scaled 0-1	Global marine plastics	[S33]
Nonindigenous species	scaled 0-1	Measure of harmful invasive species	[S37]
Genetic escapes	scaled 0-1	Introduced mariculture species (Mariculture Sustainability Index) as a proxy for genetic escapes	
Inverse Community Well-Being Index all	scaled 0-1	(1 - Community Well-Being Index) across all OHIBC census subdivisions reporting on four components and overall index	[S39]
Inverse Community Well-Being Index First Nations	scaled 0-1	(1 - Community Well-Being Index) across OHIBC First Nations census subdivisions reporting on four components and overall index	[S39]

S3 Table: Resilience layers

name	units	description	ref
Aquaculture regulations	scaled 0-1	Aquaculture-specific regulation existence, regulation enforcement, regulation compliance	[S40]
Community Well-Being Index (all)	scaled 0-1	Community Well-Being Index across all OHIBC census subdivisions reporting on four components and overall index	[S39]
Community Well-Being Index (First Nations)	scaled 0-1	Community Well-Being Index across OHIBC First Nations census subdivisions reporting on four components and overall index	[S39]
FN Res. Access Opp. component weight	scaled 0-1	Contribution of each component to the First Nations Resource Access Opportunities score	[S26]
Coastal protection weights	scaled 0-1	Habitat extent multiplied by habitat protection rank for coastal forests and saltmarsh	[S26]
Carbon storage weights	scaled 0-1	Habitat extent multiplied by carbon storage capacity for coastal forests and saltmarsh	[S26]
Habitat presence	boolean	List of habitats in each region	[S26]
Commercial fishing management	scaled 0-1	Regulations and management of commercial fishing including Fisheries Act, fisheries officers on vessels, and observer coverage	[S41,42]
Coastal protected marine areas	scaled 0-1	Protected marine areas with effective management plans within 3 nmi of coastline relative to 30% baseline to protect fishery resources	[S24]
EEZ protected marine areas	proportion	Protected marine areas with effective management plans within EEZ to protect fishery resource relative to 30% baseline to protect fishery resources	[S24]
Trawl habitat agreement	scaled 0-1	Ecosystem management based trawl reduction agreement to reduce bottom trawl impacts on deepwater corals and sponges	[S43]
Coastal protected marine areas	ratio	Protected marine areas with effective management plans within 3 nmi of coastline relative to 30% baseline to protect against habitat destruction	[S24]
Protected marine areas within EEZ	ratio	Protected marine areas with effective management plans within EEZ relative to 30% baseline to protect against habitat destruction	[S24]
MaPP Resilience	scaled 0-1	Resilience due to MaPP process and plans	[S26]
Coastal ecological integrity	scaled 0-1	Marine species condition (same calculation and data as the species subgoal status score) calculated within 3 nautical miles of shoreline as a proxy for ecological integrity	[S20-23]
Marine ecological integrity	scaled 0-1	Marine species condition (same calculation and data as the species subgoal status score) calculated within EEZ as a proxy for ecological integrity	[S20-23]

Table S4: Goal scores by region and year

Goal/subgoal	Region	2001	2002	2003	2004	2005	2006	2007	2008	2009	2010	2011	2012	2013	2014	2015	2016
Habitat Services	British Columbia	100	100	100	100	100	100	100	100	100	100	100	100	100	100	100	100
	N. Coast	100	100	100	100	100	100	100	100	100	100	100	100	100	100	100	100
	Haida Gwaii	100	100	100	100	100	100	100	100	100	100	100	100	100	100	100	100
	Central Coast	100	100	100	100	100	100	100	100	100	100	100	100	100	100	100	100
	N. Vancouver Is.	100	100	100	100	100	100	100	100	100	100	100	100	100	100	100	100
	W. Vancouver Is.	100	100	100	100	100	100	100	100	100	100	100	100	100	100	100	100
	Strait of Georgia	97	97	97	97	96	96	96	96	96	95	96	96	96	96	96	96
	Aristazabal Is.	100	100	100	100	100	100	100	100	100	100	100	100	100	100	100	100
Coastal Protection	British Columbia	100	100	100	100	100	100	100	100	100	100	100	100	100	100	100	100
	N. Coast	100	100	100	100	100	100	100	100	100	100	100	100	100	100	100	100
	Haida Gwaii	100	100	100	100	100	100	100	100	100	100	100	100	100	100	100	100
	Central Coast	100	100	100	100	100	100	100	100	100	100	100	100	100	100	100	100
	N. Vancouver Is.	100	100	100	100	100	100	100	100	100	100	100	100	100	100	100	100
	W. Vancouver Is.	100	100	100	100	100	100	100	100	100	100	100	100	100	100	100	100
	Strait of Georgia	97	96	96	96	95	95	95	94	94	94	94	94	94	94	94	94
	Aristazabal Is.	100	100	100	100	100	100	100	100	100	100	100	100	100	100	100	100
Carbon Storage	British Columbia	100	100	100	100	100	100	100	100	100	100	100	100	100	100	100	100
	N. Coast	100	100	100	100	100	100	100	100	100	100	100	100	100	100	100	100
	Haida Gwaii	100	100	100	100	100	100	100	100	100	100	100	100	100	100	100	100
	Central Coast	100	100	100	100	100	100	100	100	100	100	100	100	100	100	100	100
	N. Vancouver Is.	100	100	100	100	100	100	100	100	100	100	100	100	100	100	100	100
	W. Vancouver Is.	100	100	100	100	100	100	100	100	100	100	100	100	100	100	100	100
	Strait of Georgia	98	98	98	98	98	98	97	97	97	97	97	97	97	97	97	97
	Aristazabal Is.	100	100	100	100	100	100	100	100	100	100	100	100	100	100	100	100
Food Provision	British Columbia	51	32	36	55	54	73	81	84	74	80	75	62	54	62	71	73
	N. Coast	41	28	33	50	49	67	83	90	59	70	57	53	53	69	83	82
	Haida Gwaii	33	32	43	66	64	72	72	73	63	74	67	60	62	66	77	79
	Central Coast	72	35	31	48	48	77	89	93	86	81	76	62	47	64	61	69
	N. Vancouver Is.	57	31	31	48	48	73	87	86	85	77	89	75	48	50	58	59
	W. Vancouver Is.	69	32	30	46	46	77	87	93	88	96	90	64	45	49	58	58
	Strait of Georgia			40	71	59	71	86	92	83	97	91	75	65	81	100	100
	Aristazabal Is.	16	16	34	57	59	68	74	64	51	64	55	50	56	83	89	91
	Pacific Offshore	34	28	35	28	31	36	33	58	33	25	45	28	24	23	19	24
Wild-Capture Fisheries	British Columbia	51	32	31	37	49	75	76	76	65	62	57	48	41	41	39	43
	N. Coast	41	28	25	29	40	63	81	87	35	42	23	31	41	57	66	63
	Haida Gwaii	33	32	47	60	69	74	58	53	44	52	42	45	59	50	54	59
	Central Coast	72	35	22	24	37	83	91	94	89	65	60	49	30	48	23	37
	N. Vancouver Is.	57	31	22	24	37	74	89	81	87	57	88	75	31	18	17	17
	W. Vancouver Is.	69	32	20	22	34	82	87	94	93	95	88	53	24	16	16	16
	Aristazabal Is.	16	16	28	42	58	64	61	36	20	32	19	25	47	85	79	82
	Pacific Offshore	34	28	35	28	31	36	33	58	33	25	45	28	24	23	19	24
Aquaculture	British Columbia											51	48	38	38	52	
	Haida Gwaii											0	0	0	0	1	
	Central Coast											94	70	55	74	100	
	N. Vancouver Is.											99	99	99	97	65	
	W. Vancouver Is.											79	78	57	49	99	
	Strait of Georgia											53	67	45	27	35	

Table S4 cont'd: Goal scores by region and year

Goal/subgoal	Region	2001	2002	2003	2004	2005	2006	2007	2008	2009	2010	2011	2012	2013	2014	2015	2016
Wild-Capture Salmon	British Columbia			40	71	59	71	86	92	83	97	91	75	65	81	100	100
	N. Coast			40	71	59	71	86	92	83	97	91	75	65	81	100	100
	Haida Gwaii			40	71	59	71	86	92	83	97	91	75	65	81	100	100
	Central Coast			40	71	59	71	86	92	83	97	91	75	65	81	100	100
	N. Vancouver Is.			40	71	59	71	86	92	83	97	91	75	65	81	100	100
	W. Vancouver Is.			40	71	59	71	86	92	83	97	91	75	65	81	100	100
	Strait of Georgia			40	71	59	71	86	92	83	97	91	75	65	81	100	100
	Aristazabal Is.			40	71	59	71	86	92	83	97	91	75	65	81	100	100
First Nations Res. Access Opp.	British Columbia	72	69	76	78	71	67	62	59	66	69	75	68	71	74	71	78
	N. Coast	86	85	85	87	81	77	76	75	81	73	74	78	82	74	72	79
	Haida Gwaii	60	56	67	71	66	62	58	57	62	71	70	63	67	69	66	72
	Central Coast	58	58	70	72	65	61	57	51	58	56	62	55	54	66	60	67
	N. Vancouver Is.	84	78	83	85	76	72	67	58	66	71	78	70	76	82	79	87
	W. Vancouver Is.	92	87	89	87	76	72	62	59	73	74	91	79	79	83	82	93
	Strait of Georgia	61	59	72	72	66	61	56	56	60	65	75	71	70	78	71	78
	Aristazabal Is.	66	66	73	78	70	64	57	56	63	68	67	63	65	60	68	76
Coastal Livelihoods	British Columbia	66	64	63	61	60	59	60	62	64	66	67	68	69	70	70	71
	N. Coast	53	52	50	49	48	46	50	54	59	64	66	67	67	67	68	69
	Haida Gwaii	67	66	64	62	61	60	61	63	65	67	68	69	70	71	71	71
	Central Coast	71	69	67	66	64	63	64	65	65	65	64	65	67	70	72	74
	N. Vancouver Is.	65	64	62	60	59	58	59	60	61	63	63	65	67	69	71	73
	W. Vancouver Is.	67	65	63	62	60	58	61	63	67	69	71	71	70	69	68	68
	Strait of Georgia	73	72	70	69	68	67	67	66	66	67	67	69	71	74	75	76
First Nations Livelihoods	British Columbia	66	64	63	61	60	59	60	62	64	66	67	68	69	70	70	71
	N. Coast	53	52	50	49	48	46	50	54	59	64	66	67	67	67	68	69
	Haida Gwaii	67	66	64	62	61	60	61	63	65	67	68	69	70	71	71	71
	Central Coast	71	69	67	66	64	63	64	65	65	65	64	65	67	70	72	74
	N. Vancouver Is.	65	64	62	60	59	58	59	60	61	63	63	65	67	69	71	73
	W. Vancouver Is.	67	65	63	62	60	58	61	63	67	69	71	71	70	69	68	68
	Strait of Georgia	73	72	70	69	68	67	67	66	66	67	67	69	71	74	75	76
Non-First Nations Livelihoods	British Columbia	89	88	88	87	87	86	86	87	88	88	89	89	89	89	89	88
	N. Coast	93	93	93	92	92	91	91	90	90	90	89	92	95	98	99	100
	Haida Gwaii	90	88	86	85	83	81	82	83	84	85	86	87	86	85	84	84
	Central Coast	83	83	82	82	82	81	83	85	87	89	89	86	83	80	76	74
	N. Vancouver Is.	88	88	88	88	88	87	88	88	89	89	89	89	90	90	90	91
	W. Vancouver Is.	88	89	89	90	91	91	91	92	91	91	92	92	93	94	95	95
	Strait of Georgia	91	92	92	92	92	92	92	92	93	93	93	94	95	96	97	98
Tourism & Recreation	British Columbia							100	100	97	92	86	89	89	96	99	95
	N. Coast							100	100	100	81	77	73	60	92	100	100
	Haida Gwaii							99	100	99	92	80	86	94	98	100	100
	N. Vancouver Is.							100	100	98	96	92	96	100	99	98	80
	W. Vancouver Is.							100	99	92	97	99	100	92	90	99	89
	Strait of Georgia							100	99	100	97	86	90	99	99	100	100

Table S4 cont'd: Goal scores by region and year

Goal/subgoal	Region	2001	2002	2003	2004	2005	2006	2007	2008	2009	2010	2011	2012	2013	2014	2015	2016
Sense of Place	British Columbia	51	51	52	52	50	55	56	68	68	69	68	67	67	67	67	67
	N. Coast	34	34	34	37	37	51	52	61	61	60	57	54	54	54	54	54
	Haida Gwaii	54	54	54	54	54	54	54	81	81	83	83	83	83	83	83	83
	Central Coast	38	38	38	38	38	53	59	76	76	76	76	70	70	70	70	70
	N. Vancouver Is.	40	40	40	40	40	46	50	51	51	48	47	46	46	46	46	46
	W. Vancouver Is.	74	74	74	74	64	64	64	64	64	64	65	65	65	65	64	64
	Strait of Georgia	43	43	45	45	45	44	43	42	42	42	43	43	43	43	43	43
	Aristazabal Is.	65	65	65	65	65	65	65	65	65	65	65	65	65	65	65	65
	Pacific Offshore	66	66	66	66	66	66	66	66	66	66	66	66	66	66	66	66
Iconic Species	British Columbia	66	66	66	66	66	66	66	66	66	66	66	66	66	66	66	66
	N. Coast	65	65	65	65	65	65	65	65	65	65	65	65	65	65	65	65
	Haida Gwaii	65	65	65	65	65	65	65	65	65	65	65	65	65	65	65	65
	Central Coast	65	65	65	65	65	65	65	65	65	65	65	65	65	65	65	65
	N. Vancouver Is.	66	66	66	66	66	66	66	66	66	66	66	66	66	66	66	66
	W. Vancouver Is.	66	66	66	66	66	66	66	66	66	66	66	66	66	66	66	66
	Strait of Georgia	66	66	66	66	66	66	66	66	66	66	66	66	66	66	66	66
	Aristazabal Is.	65	65	65	65	65	65	65	65	65	65	65	65	65	65	65	65
	Pacific Offshore	66	66	66	66	66	66	66	66	66	66	66	66	66	66	66	66
Lasting Special Places	British Columbia	37	37	37	38	34	43	46	71	72	72	72	69	69	69	69	69
	N. Coast	3	3	3	8	8	36	38	57	57	54	49	43	43	43	43	43
	Haida Gwaii	43	43	43	43	43	43	43	96	96	100	100	100	100	100	100	100
	Central Coast	11	11	11	11	11	41	52	88	88	88	88	75	75	75	75	75
	N. Vancouver Is.	13	13	13	13	13	26	35	35	36	29	27	27	27	27	27	27
	W. Vancouver Is.	81	81	81	81	63	63	62	62	62	62	63	63	63	63	63	63
	Strait of Georgia	21	21	24	24	23	22	19	18	18	18	20	20	21	20	20	20
Biodiversity	British Columbia	94	94	94	94	93	93	92	91	91	92	93	92	92	93	92	92
	N. Coast	92	92	92	92	92	92	92	89	91	92	92	92	92	92	92	92
	Haida Gwaii	94	94	94	94	94	94	91	91	90	90	92	92	92	93	93	93
	Central Coast	94	94	94	94	94	94	90	88	90	90	92	92	91	91	91	92
	N. Vancouver Is.	93	93	93	93	93	93	93	93	93	93	93	93	93	93	93	93
	W. Vancouver Is.	95	95	95	95	95	95	95	95	95	95	95	95	95	95	95	95
	Strait of Georgia	86	86	86	86	86	88	85	84	86	89	89	87	83	90	84	84
	Aristazabal Is.	93	93	93	93	93	93	93	93	93	93	93	93	93	93	93	93
	Pacific Offshore	96	96	96	96	96	96	96	96	96	96	96	96	96	96	96	96
Species	British Columbia	88	88	88	88	88	88	88	88	88	88	88	88	88	88	88	88
	N. Coast	85	85	85	85	84	84	85	85	85	85	85	85	85	85	85	85
	Haida Gwaii	89	89	89	89	88	88	88	89	89	89	89	89	89	89	89	89
	Central Coast	89	89	88	88	88	88	88	88	88	88	88	88	89	89	89	89
	N. Vancouver Is.	87	87	87	87	87	87	87	87	87	87	87	87	87	87	87	87
	W. Vancouver Is.	90	90	90	90	90	90	90	90	90	90	90	90	90	90	90	90
	Strait of Georgia	85	85	85	85	85	85	85	85	85	85	85	85	85	85	85	85
	Aristazabal Is.	87	86	86	86	86	86	86	86	86	86	86	86	86	86	87	86
	Pacific Offshore	91	91	92	92	91	92	92	93	93	93	93	93	93	93	93	93

Table S4 cont'd: Goal scores by region and year

Goal/subgoal	Region	2001	2002	2003	2004	2005	2006	2007	2008	2009	2010	2011	2012	2013	2014	2015	2016
	British Columbia	99	99	99	99	99	99	95	94	94	96	97	97	96	98	97	97
	N. Coast	100	100	100	100	100	100	99	94	98	100	100	100	98	99	99	100
	Haida Gwaii	100	100	100	100	100	100	94	93	91	92	95	95	96	97	97	97
	Central Coast	100	100	100	100	100	100	92	87	91	92	96	95	94	94	94	95
Habitats	N. Vancouver Is.	99	99	99	99	99	98	98	98	98	98	98	98	98	98	98	98
	W. Vancouver Is.	100	100	100	100	100	100	100	100	100	100	100	100	100	100	100	100
	Strait of Georgia	87	88	88	88	88	91	86	83	87	94	94	89	81	95	83	83
	Aristazabal Is.	100	100	100	100	100	100	99	99	100	100	100	100	100	100	100	100
	Pacific Offshore	100	100	100	100	100	100	100	100	100	100	100	100	100	100	100	100
	British Columbia	88	88	88	88	88	88	88	88	88	88	88	88	88	88	88	88
	N. Coast	87	87	87	87	87	87	87	87	87	87	87	87	87	87	87	87
	Haida Gwaii	91	91	91	91	91	91	91	91	91	91	91	91	91	91	91	91
Clean Waters	Central Coast	87	87	87	87	87	87	87	87	87	87	87	87	87	87	87	87
	N. Vancouver Is.	89	89	89	89	89	89	89	89	89	89	89	89	89	89	89	89
	W. Vancouver Is.	85	85	86	86	86	86	85	86	86	85	84	85	86	86	86	85
	Strait of Georgia	82	82	82	82	82	82	82	82	82	82	82	82	82	82	82	82
	Aristazabal Is.	86	86	86	86	86	86	86	86	86	86	86	86	86	86	86	86
	British Columbia	75	71	73	75	74	76	79	81	81	82	81	79	78	81	82	83
	N. Coast	71	68	69	72	71	74	80	82	80	78	76	75	74	79	82	83
	Haida Gwaii	72	71	73	77	76	76	78	82	81	84	81	80	82	84	85	86
	Central Coast	74	69	70	72	71	77	78	80	80	79	80	76	74	78	77	80
Index	N. Vancouver Is.	75	71	71	74	72	76	81	80	80	80	81	79	77	79	79	78
	W. Vancouver Is.	83	77	77	78	75	79	82	82	83	85	87	82	79	80	82	82
	Strait of Georgia	74	73	70	75	72	73	77	77	77	79	79	77	76	80	81	82
	Aristazabal Is.	71	71	75	80	79	79	79	77	76	79	78	76	77	81	84	85
	Pacific Offshore	65	63	66	63	64	66	65	74	65	62	69	64	62	62	61	62

Table S5: Changes in goal scores over time (all BC)

goal	intercept	year	adj.R^2
Habitat Services	99.7656***	-0.0078***	0.717
Food Provision	50.6812***	1.7313*	0.222
First Nations Res. Access Opp.	69.6151***	0.0968	-0.064
Coastal Livelihoods	60.4177***	0.6215**	0.507
Tourism & Recreation	98.0817***	-0.3593	-0.070
Sense of Place	50.2633***	1.4307***	0.709
Biodiversity	93.2798***	-0.0934°	0.183
Clean Waters	87.9947***	-0.0068	-0.021
Index	73.3682***	0.6485***	0.696

Significance codes: ***: $p < 0.001$; **: $p < 0.01$; *: $p < 0.05$; °: $p < 0.1$

Table S6: Changes in goal scores over time (by region)

goal	region	intercept	year	adj.R^2
Habitat Services	North Coast	99.9845***	-0.0001***	0.573
	Haida Gwaii	99.9906***	0.0002**	0.495
	Central Coast	99.9955***	-0.0002***	0.722
	N. Vancouver Is.	99.8061***	-0.0071***	0.693
	W. Vancouver Is.	99.8722***	-0.0041***	0.710
	Strait of Georgia	96.9189***	-0.1056***	0.721
	Aristazabal Is.	100.0000***	0.0000	NaN
Food Provision	North Coast	42.3479***	2.4073**	0.349
	Haida Gwaii	47.1153***	2.0870**	0.442
	Central Coast	57.2326***	1.0334	0.001
	N. Vancouver Is.	53.7555***	1.1922	0.020
	W. Vancouver Is.	57.9523***	0.8302	-0.037
	Strait of Georgia	57.0000***	2.6279*	0.364
	Aristazabal Is.	30.8218***	3.6096***	0.571
First Nations Res. Access Opp.	North Coast	84.3596***	-0.7079**	0.428
	Haida Gwaii	61.0622***	0.5077°	0.159
	Central Coast	61.7977***	-0.1679	-0.053
	N. Vancouver Is.	75.9625***	-0.0308	-0.071
	W. Vancouver Is.	80.0082***	-0.0203	-0.071
	Strait of Georgia	60.3194***	0.8722*	0.259
	Aristazabal Is.	67.1096***	-0.1122	-0.063
Coastal Livelihoods	North Coast	46.1729***	1.5803***	0.769
	Haida Gwaii	61.9169***	0.5548**	0.452
	Central Coast	65.4839***	0.1895	0.005
	N. Vancouver Is.	59.1981***	0.6166**	0.405
	W. Vancouver Is.	61.5932***	0.5560**	0.373
	Strait of Georgia	67.7980***	0.2593	0.072
Tourism & Recreation	North Coast	95.0607***	-0.6451	-0.105
	Haida Gwaii	93.7252***	0.1024	-0.123
	N. Vancouver Is.	106.7987***	-1.0392	0.181
	W. Vancouver Is.	103.3913***	-0.7324	0.162
	Strait of Georgia	96.1618***	0.0811	-0.122
Sense of Place	North Coast	36.9151***	1.6300***	0.539
	Haida Gwaii	50.3406***	2.6188***	0.748
	Central Coast	38.7392***	2.8385***	0.659
	N. Vancouver Is.	41.1346***	0.5396*	0.341
	W. Vancouver Is.	71.3979***	-0.6259**	0.498
	Strait of Georgia	43.9960***	-0.0816°	0.132
	Aristazabal Is.	65.2193***	-0.0000	0.478

Table S6 cont'd: Changes in goal scores over time (by region)

goal	region	intercept	year	adj.R^2
Biodiversity	North Coast	91.9547***	-0.0074	-0.069
	Haida Gwaii	93.8359***	-0.1601°	0.179
	Central Coast	93.8321***	-0.2374*	0.264
	N. Vancouver Is.	93.0777***	-0.0264*	0.253
	W. Vancouver Is.	94.8511***	0.0073**	0.391
	Strait of Georgia	86.5767***	-0.0369	-0.063
	Aristazabal Is.	93.1223***	-0.0002	-0.071
Clean Waters	North Coast	87.4109***	-0.0151**	0.424
	Haida Gwaii	90.8960***	0.0042	-0.035
	Central Coast	87.0329***	-0.0034	0.065
	N. Vancouver Is.	88.9883***	-0.0027	-0.035
	W. Vancouver Is.	85.8549***	-0.0316	-0.015
	Strait of Georgia	81.9397***	0.0163	0.036
	Aristazabal Is.	85.6178***	-0.0018**	0.380
Index	North Coast	70.0141***	0.7900***	0.564
	Haida Gwaii	72.2289***	0.9409***	0.878
	Central Coast	72.0163***	0.5218**	0.379
	N. Vancouver Is.	73.2533***	0.5060**	0.442
	W. Vancouver Is.	78.8373***	0.2553	0.091
	Strait of Georgia	71.6061***	0.6354***	0.733
	Aristazabal Is.	73.6485***	0.5826**	0.505

Significance codes: ***: $p < 0.001$; **: $p < 0.01$; *: $p < 0.05$; °: $p < 0.1$

Table S7: Proportional change in pressure (at t + λ) vs. resilience (at t), all lag years
Fixed effect coefficients on region and year are omitted for clarity.

goal	subgoal	λ	intercept	reg resil	soc resil	adj.R²	RMSE
Habitat Services	Coastal Protection	1	0.4048*	-0.0795*	-0.7165*	0.639	0.0312
		2	0.7100*	-0.1394*	-1.2586*	0.697	0.0521
		3	0.8768*	-0.1653°	-1.5391*	0.738	0.0644
		4	0.7556	-0.1486	-1.3913°	0.775	0.0700
		5	-0.0149	-	-0.2610	0.796	0.0741
		6	-0.2359	-	-0.0086	0.823	0.0737
	Carbon Storage	1	0.4048*	-0.0795*	-0.7165*	0.639	0.0312
		2	0.7100*	-0.1394*	-1.2586*	0.697	0.0521
		3	0.8768*	-0.1653°	-1.5391*	0.738	0.0644
		4	0.7556	-0.1486	-1.3913°	0.775	0.0700
		5	-0.0149	-	-0.2610	0.796	0.0741
		6	-0.2359	-	-0.0086	0.823	0.0737
Food Provision	Wild-Capture Fisheries	1	-0.0438*	-0.2209*	-	0.509	0.0414
		2	-0.1194***	-0.4423***	-	0.667	0.0540
		3	-0.7030***	-	0.8907**	0.662	0.0610
		4	-0.8998***	-	1.2195***	0.671	0.0677
		5	-1.2763***	-	1.7883***	0.719	0.0660
		6	-1.5178***	-	2.1242***	0.727	0.0639
	Aquaculture	1	-0.0600**	-	-	0.611	0.0495
		2	-0.1183***	-	-	0.804	0.0510
		3	-0.0334	-	-	0.802	0.0653
		4	0.0203	-	-	0.833	0.0682
		5	0.0891**	-	-	0.838	0.0732
		6	0.1056***	-	-	0.874	0.0623
	Wild-Capture Salmon	1	0.0013	-0.1237°	-	0.447	0.0238
		2	-0.0053	-0.2475*	-	0.586	0.0338
		3	-0.4193**	-	0.4906*	0.632	0.0395
		4	-0.4665**	-	0.5797*	0.610	0.0453
		5	-0.6085**	-	0.7850**	0.625	0.0470
		6	-0.7456***	-	0.9672**	0.644	0.0456
First Nations Res. Access Opp.		1	-0.2188	-	0.2381	0.330	0.0636
		2	-0.5321	-	0.7934	0.515	0.0838
		3	-0.8113°	-	1.3125	0.665	0.0867
		4	-1.1822*	-	2.0034*	0.716	0.0923
		5	-1.6312**	-	2.7867**	0.766	0.0894
		6	-2.1721***	-	3.6771***	0.813	0.0785

Table S7 (cont'd): Proportional change in pressure (at t + λ) vs. resilience (at t), all lag years

Fixed effect coefficients on region and year are omitted for clarity.

goal	subgoal	λ	intercept	reg resil	soc resil	adj.R²	RMSE
Tourism & Recreation		2	0.1171	-	-0.2329	0.656	0.0358
		3	0.2375°	-	-0.3903°	0.691	0.0409
		4	0.3762**	-	-0.5636*	0.715	0.0409
		5	0.5746**	-	-0.8488**	0.651	0.0465
		6	0.5527**	-	-0.7951**	0.709	0.0419
		1	-0.0227°	-	-	0.496	0.0284
Sense of Place	Iconic Species	4	-0.4288	1.2322	0.1517	0.528	0.1183
		5	0.0142	1.2170	-0.5166	0.553	0.1268
		6	1.0698°	-	-1.7466*	0.570	0.1242
		1	-0.0063	-	-	0.218	0.0733
		2	-0.0246	-	-	0.201	0.1025
		3	-0.0174	-	-	0.297	0.1153
	Lasting Special Places	2	0.0772	-	-0.1590°	0.524	0.0199
		3	0.1797*	-	-0.3162**	0.533	0.0230
		4	0.2427**	-	-0.4071***	0.640	0.0214
		5	0.2789**	-	-0.4694**	0.645	0.0244
		6	0.2622*	-	-0.4383*	0.714	0.0251
		1	-0.0101	-	-	0.316	0.0185
Biodiversity	Species	1	-0.0783	-	0.0779	0.491	0.0320
		2	-0.2192°	-	0.2329	0.615	0.0394
		3	-0.2319	-	0.2533	0.620	0.0457
		4	-0.2728°	-	0.3411	0.661	0.0474
		5	-0.3579°	-	0.4832°	0.673	0.0493
		6	-0.5230*	-	0.6721*	0.661	0.0472
	Habitats	4	-0.8306°	0.6710	0.9065	0.429	0.0644
		6	-1.0909*	0.7083	1.2708*	0.632	0.0559
		3	-0.1579	-	0.1125	0.368	0.0626
		5	-0.4082°	-	0.4967	0.490	0.0641
		1	-0.0277	-	-	0.244	0.0496
		2	-0.0936***	-	-	0.402	0.0539
Clean Waters		1	-0.0014	0.0026	-	0.124	0.0191
		3	-0.0632	0.0751	-	0.120	0.0229
		1	0.0004	-	-	0.124	0.0191
		2	-0.0106	-	-	0.107	0.0220
		3	-0.0118	-	-	0.120	0.0229
		4	-0.0066	-	-	0.235	0.0201
		5	-0.0127	-	-	0.238	0.0243
		6	0.0028	-	-	0.416	0.0206

Significance codes: ***: $p < 0.001$; **: $p < 0.01$; *: $p < 0.05$; °: $p < 0.1$

Table S8: Proportional change in status (at t + λ) vs. pressure (at t), all lag years

Fixed effect coefficients on region and year are omitted for clarity.

goal	subgoal	λ	intercept	pressures	adj.R²	RMSE
Habitat Services	Coastal Protection	1	-0.0006°	0.0001**	0.582	0.0007
		2	-0.0011°	0.0002**	0.601	0.0013
		3	-0.0016°	0.0003*	0.621	0.0020
		4	-0.0018	0.0003*	0.645	0.0025
		5	-0.0020	0.0003°	0.677	0.0030
		6	-0.0020	0.0003	0.721	0.0033
	Carbon Storage	1	-0.0001°	0.0000*	0.580	0.0002
		2	-0.0002°	0.0000*	0.596	0.0003
		3	-0.0004	0.0001°	0.617	0.0005
		4	0.0001	-0.0001	0.640	0.0006
		5	0.0002	-0.0002°	0.682	0.0007
		6	0.0003	-0.0003*	0.734	0.0008
Food Provision	Wild-Capture Fisheries	1	-0.0699	-	0.076	0.2843
		2	0.1668	-	0.173	0.4223
		3	0.4159°	-	0.238	0.5601
		4	0.6621*	-	0.275	0.6383
		5	-0.9209	0.1426	0.281	0.6915
		6	-1.4713	0.1848°	0.310	0.6353
	Aquaculture	1	-0.6263	-	0.144	2.7203
		2	-0.2589	-	0.432	1.2741
		3	-1.4660	-	0.046	6.8779
	Wild-Capture Salmon	1	0.7732***	-0.0000	1.000	0.0000
		2	0.4682***	0.0000	1.000	0.0000
		3	0.7677***	0.0000	1.000	0.0000
		4	0.7746***	0.0000	1.000	0.0000
		5	1.1018***	-0.0000°	1.000	0.0000
		6	0.8297***	0.0000	1.000	0.0000
First Nations Res. Access Opp.		1	0.0006	-	0.486	0.0565
		2	0.3027**	-0.0120°	0.575	0.0679
		3	0.1145***	-	0.660	0.0654
		4	0.0202	-	0.664	0.0684
		5	0.2034	-0.0132	0.634	0.0710
		6	-0.0611°	-	0.630	0.0783

Table S8 cont'd: Proportional change in status (at t + λ) vs. pressure (at t), all lag years

Fixed effect coefficients on region and year are omitted for clarity.

goal	subgoal	λ	intercept	pressures	adj.R²	RMSE
Tourism & Recreation		1	-0.4966	0.0180	-0.038	0.0972
		2	-0.8094°	0.0340°	0.070	0.1358
		3	-1.2112	0.0418	0.052	0.1546
		4	-1.4845°	0.0521°	0.212	0.1315
		5	-0.0444	-	0.088	0.1415
		6	-0.0653	-	-0.130	0.1458
Sense of Place	Lasting Special Places	1	1.7810	-0.0803	0.212	0.4568
		2	3.3316	-0.1468	0.310	0.6550
		3	6.2391	-0.2655	0.282	1.4058
		4	9.8594	-0.4252	0.333	1.8645
		5	16.3578	-0.6424	0.382	2.4470
		6	27.1162	-1.1226	0.424	2.7952
Biodiversity	Species	1	-0.0007***	-	0.781	0.0005
		2	-0.0013***	-	0.769	0.0007
		3	-0.0014***	-	0.785	0.0009
		4	-0.0031***	-	0.782	0.0010
		5	-0.0031***	-	0.759	0.0010
		6	-0.0045*	0.0001	0.744	0.0010
	Habitats	1	0.0009	-	0.044	0.0245
		2	-0.0270	0.0027	0.132	0.0268
		3	-0.0403	0.0040°	0.213	0.0241
		4	-0.0632*	0.0063**	0.252	0.0233
		5	-0.0911**	0.0092**	0.252	0.0269
		6	-0.1503***	0.0129***	0.354	0.0289
Clean Waters		1	-0.0789***	0.0025***	0.423	0.0028
		2	-0.0928***	0.0031***	0.465	0.0029
		3	-0.0938***	0.0031***	0.454	0.0031
		4	-0.0704***	0.0023***	0.359	0.0032
		5	-0.1035***	0.0034***	0.507	0.0033
		6	-0.0854***	0.0028***	0.352	0.0031

Significance codes: ***: p < 0.001; **: p < 0.01; *: p < 0.05; °: p < 0.1

Supporting Methods

Supporting Methods: Goal models and data

Habitat Services

The Habitat Services score is the average of Coastal Protection (CPP) and Carbon Storage (CSS) subgoals:

$$X_{rgn,yr}^{HS} = \frac{1}{2}(X_{rgn,yr}^{CPP} + X_{rgn,yr}^{CSS})$$

The CPP and CSS subgoals are described below.

Coastal Protection

To determine coastal protection within a region, we sum the protective potential of each spatialized unit of coastal habitat based on the protective value of that habitat type and the exposure of the habitat site, and compare this total protective potential to that of an historic baseline.

Habitat extent for coastal forests A_{cf} and salt marsh A_{sm} are based on 30 m land use rasters [1], clipped to forest and marsh habitat within 1 km of the shoreline and no more than 5 m elevation. Coastal exposure for a given cell E_{cell} is based on exposure classes from the British Columbia Marine Conservation Analysis project (BCMCA) [2]; raw values from 1 ("highly protected") to 6 ("highly exposed"), are rescaled from 0 to 1. Protection weights for habitat types are based on vulnerability values from InVEST Coastal Vulnerability Model[3]:

Vulnerability	Very Low	Low	Moderate	High	Very High
Score	1	2	3	4	5
Natural Habitats	Coral reef; mangrove; coastal forest	High dune; marsh	Low dune	Seagrass; kelp	No habitat

Protective capacity weights for coastal forest w_{cf} and salt marsh w_{sm} are calculated as (1 - Vulnerability) / 4, i.e. rescaled 0 to 1.

A region's score is based on protective capacity-weighted total exposure $E_{rgn,yr}$ relative to a reference condition $E_{rgn,ref}$ in 1990.

$$X_{rgn,yr}^{CPP} = \min\left[w_{cf}\left(\frac{E_{rgn,yr}^{cf}}{E_{rgn,ref}^{cf}}\right) + w_{sm}\left(\frac{E_{rgn,yr}^{sm}}{E_{rgn,ref}^{sm}}\right), \quad 1\right]$$

Coastal forest and salt marsh exposure are exposure-weighted area of each habitat, based on 30 m land use rasters and exposure class of each cell:

$$E_{rgn,yr}^{sm} = (.03km)^2 \sum_{cell=1}^{n} \mathbb{1}_{[\text{cell = salt marsh}]} \times E_{cell}$$

$$E_{rgn,yr}^{cf} = (.03km)^2 \sum_{cell=1}^{n} \mathbb{1}_{[\text{cell = coastal forest}]} \times E_{cell}$$

Gapfilling: Since land use rasters were available only for 1990, 2000, and 2010, values for intervening years are based on linear interpolation, e.g. $x_{2004} = 0.6x_{2000} + 0.4x_{2010}$. For values after 2010, the 2010 value is carried forward.

Carbon Storage

Carbon storage potential is scored based on the current extent of all carbon sequestering habitats, weighted by the amount of carbon effectively sequestered in a unit of each habitat. Scores for this goal compare carbon storage potential to an historic baseline.

As in the Coastal Protection subgoal, habitat extent for coastal forests A_{cf} and salt marsh A_{sm} are based on 30 m land use rasters [1]. For salt marsh, all wetland cells within 1 km of the shoreline are included. For coastal forests, we included all forest cells found within sub-watersheds incident with the coastline, and did not consider elevation.

Carbon sequestration potential is based on carbon burial rates c for each habitat, measured in gC m^{-2} yr^{-1} [4].

- Salt marsh c_{sm}: 218 +/- 24 gC m^{-2} yr^{-1} (mean +/- SE)
- Coastal boreal forests c_{cf}: 4.6 +/- 2.1 gC m^{-2} yr^{-1}

$$X_{rgn,yr}^{CS} = \min\left(\frac{c_{cf}A_{rgn,yr}^{cf} + c_{sm}A_{rgn,yr}^{sm}}{c_{cf}A_{rgn,ref}^{cf} + c_{sm}A_{rgn,ref}^{sm}},\quad 1 \right)$$

Coastal forest and salt marsh area are based on number of cells for that habitat in 30 m land use rasters within the appropriate buffer zone:

$$A_{rgn,yr}^{sm} = (.03km)^2 \times n_{cells,saltmarsh}$$

$$A_{rgn,yr}^{cf} = (.03km)^2 \times n_{cells,forest}$$

Gapfilling: Since land use rasters were available only for 1990, 2000, and 2010, values for other years were gap filled in the same manner as for the Coastal Protection subgoal.

Food Provision

The Food Provision goal is calculated as the sum of wild-capture fisheries, aquaculture, and wild-capture salmon subgoals divided by the number of non-NA subgoals available for that region and year.

$$X_{rgn,yr}^{FP} = \frac{1}{n_{rgn,yr}^{sub}} \sum_{sub \in FIS,MAR,SAL} X_{rgn,yr}^{sub}$$

The wild-capture fisheries, aquaculture, and wild-capture salmon subgoals are described below.

Wild-Capture Fisheries

Wild-capture fisheries are scored as a catch-weighted average of the health and management status of all stocks assessed against an MSY reference point (i.e. those with reported catch per DFO [6] and either B/B_{MSY} or F/F_{MSY} or both in the RAM database [35], hereafter "assessed," vs. "unassessed" stocks lacking an MSY reference point) within a region, modified by a penalty to account for unassessed stocks targeted within the region. Spatially explicit landings information for 20 species, representing 47 different stocks (S9 Table), for the years 2007 to 2015 were provided by DFO [6]. Scores for assessed fishery stocks are based on both the total biomass of the stock B relative to biomass at maximum sustainable yield (MSY), B_{MSY}, and the fishing mortality F relative to that at MSY, F_{MSY}, as reported by the RAM Legacy database [5]. These B/B_{MSY} and F/F_{MSY} values are rescaled from 0 to 1 based on a goal of maximizing sustainable yield, resulting in $F' \in [0,1]$ and $B' \in [0,1]$ (S5 Fig.). Unassessed stocks are given a score of half the catch-weighted average score of the region's assessed stocks. The overall score for a given region and year is the catch-weighted mean of all assessed and unassessed stocks within a region for that year.

$$X_{rgn,yr}^{FIS,assessed} = \frac{\sum_{stock=1}^{n} F'_{stock} \times B'_{stock} \times C_{stock}}{\sum_{stock=1}^{n} C_{stock}}$$

$$X_{rgn,yr}^{FIS,unassessed} = 0.5 \times X_{rgn,yr}^{FIS,assessed}$$

$$X_{rgn,yr}^{FIS} = \frac{X_{rgn,yr}^{FIS,assessed} \times \sum C_{stock,assessed} + X_{rgn,yr}^{FIS,unassessed} \times \sum C_{stock,unassessed}}{\sum C_{stock,assessed} + \sum C_{stock,unassessed}}$$

Note F/F_{MSY} data were unavailable for some assessed stocks, in which case the stock score was based on the B' term.

See S9 Table for a list of all OHIBC stocks including assessment status.

Rescaling B/B_{MSY}

Rescaled biomass score B' for each stock is calculated based on B/B_{MSY}, with a score of 1 indicating B/B_{MSY} near 1.0, decreasing to 0 as B/B_{MSY} approaches 0 (overfished), with an increasing penalty for B/B_{MSY} above 1.5 (underfished), with a minimum underfished score of 0.25 for $B/B_{MSY} \geq 3.0$.

$$B' = \begin{cases} \frac{B/B_{MSY}}{0.8} & \text{when } B/B_{MSY} < 0.8 \\ 1 & \text{when } 0.8 \geq B/B_{MSY} < 1.5 \\ 1.75 - \frac{1}{2}B/B_{MSY} & \text{when } 1.5 \geq B/B_{MSY} < 3.0 \\ 0.25 & \text{when } B/B_{MSY} \geq 3.0 \end{cases}$$

Rescaling F/F_{MSY}

Rescaled fishing mortality F' for each stock is calculated based on F/F_{MSY}, smoothed using a rolling four-year mean. A DFO harvest control rule indicates no targeted catch for B/B_{MSY} below a critical threshold of 0.4, increasing to $F/F_{MSY} = 1$ for $B/B_{MSY} \geq 0.8$. Our calculation allows a buffer around this to account for uncertainty in setting annual management targets, and incorporates an overfishing penalty ($F' = 0$ for $F/F_{MSY} \geq 2$) as well as an underfishing penalty to account for lost opportunity for additional sustainable catch.

When $B/B_{MSY} \geq 0.8$ (healthy stock):

$$F' = \begin{cases} 0 & \text{when } F/F_{MSY} \geq 2.0 \\ 2.5 - 1.25F/F_{MSY} & \text{when } 2.0 \geq F/F_{MSY} \geq 1.2 \\ 1 & \text{when } 1.2 \geq F/F_{MSY} \geq 0.8 \\ 0.25 + 0.6F/F_{MSY} & \text{when } 0.8 \geq F/F_{MSY} \geq 0 \end{cases}$$

When $B/B_{MSY} < 0.8$ (overexploited stock):

$$F' = \begin{cases} 0 & \text{when } F/F_{MSY} - 2.5B/B_{MSY} \geq 0 \\ 2.0 + F/F_{MSY} - 2.5B/B_{MSY}) & \text{when } 0 \geq F/F_{MSY} - 2.5B/B_{MSY} \geq -0.8 \\ 1 & \text{when } -0.8 \geq F/F_{MSY} - 2.5B/B_{MSY} \geq -1.2 \\ 0.25 + 0.6(F/F_{MSY} + 2.5B/B_{MSY}) & \text{when } 0.8 \geq F/F_{MSY} - 2.5B/B_{MSY} \geq 0 \end{cases}$$

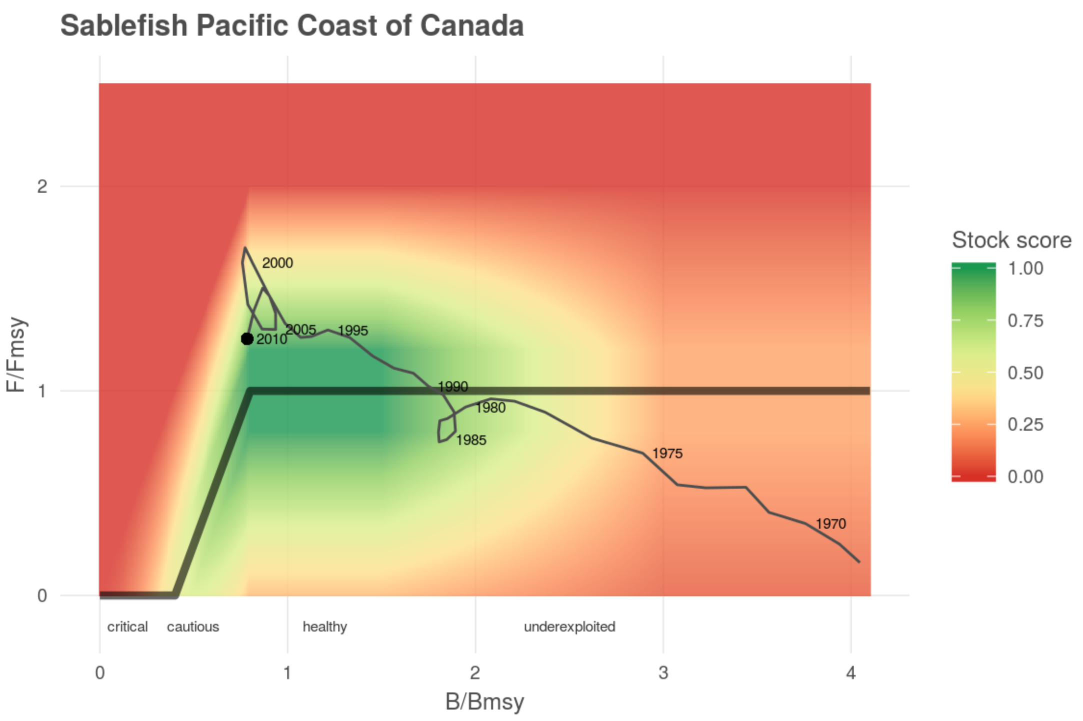

Figure S5: Modified Kobe plot showing scoring for F/F_{MSY} and B/B_{MSY}. Sablefish scores over time are shown as an example of stock status scoring changes over time.

S9 Table: Stocks included in OHIBC Wild-Capture Fisheries assessment

Stock ID	Stock description	Scientific name	Common name	Assessed?*
ALBANPAC	Albacore tuna N. Pac.	Thunnus alalunga	Albacore tuna	yes
BOCACCBCW	Bocaccio BC Waters	Sebastes paucispinis	Bocaccio	yes
LINGCODSOG	Lingcod Str. of Georgia	Ophiodon elongatus	Lingcod	yes
PCODHS	Pacific cod Hecate Strait	Gadus macrocephalus	Pacific cod	yes
PERCHQCI	Pacific Ocean perch Haida Gwaii	Sebastes alutus	Pacific Ocean perch	yes
PERCHWCVANI	Pacific Ocean perch W. Coast Van. Is.	Sebastes alutus	Pacific Ocean perch	yes
PHAKEPCOAST	Pacific hake Pac. Coast	Merluccius productus	Pacific hake	yes
PHALNPAC	Pacific halibut N. Pac.	Hippoglossus stenolepis	Pacific halibut	yes
RSOLE5AB	Rock sole Queen Charlotte Sound	Lepidopsetta bilineata	Rock sole	yes
RSOLEHSTR	Rock sole Hecate Strait	Lepidopsetta bilineata	Rock sole	yes
SABLEFPCAN	Sablefish Pac. Coast Canada	Anoplopoma fimbria	Sablefish	yes
BIGSKA3CD	Big skate W. Coast Van. Is.	Raja binoculata	Big skate	no
BIGSKA4B	Big skate Str. of Georgia	Raja binoculata	Big skate	no
BIGSKA5AB	Big skate Queen Charlotte Sound	Raja binoculata	Big skate	no
BIGSKA5CDE	Big skate Hecate Strait	Raja binoculata	Big skate	no
CROCKWCVANISOG QCI	Canary rockfish W. Coast Van. Is., Str. of Georgia, Queen Charlotte Is.	Sebastes pinniger	Canary rockfish	no
ESOLEHS	English sole Hecate Strait	Parophrys vetulus	English sole	no
EULAPCOASTCCDU	Eulachon Pac. Coast Central Coast DU	Thaleichthys pacificus	Eulachon	no

S9 Table: Stocks included in OHIBC Wild-Capture Fisheries assessment

Stock ID	Stock description	Scientific name	Common name	Assessed?*
EULAPCOASTFRDU	Eulachon Pac. Coast Fraser River DU	Thaleichthys pacificus	Eulachon	no
EULAPCOASTNSDU	Eulachon Pac. Coast Nass / Skeena DU	Thaleichthys pacificus	Eulachon	no
HERRCC	Pacific herring Central Coast	Clupea pallasii	Pacific herring	no
HERRPRD	Pacific herring Prince Rupert District	Clupea pallasii	Pacific herring	no
HERRQCI	Pacific herring Haida Gwaii	Clupea pallasii	Pacific herring	no
HERRSOG	Pacific herring Str. of Georgia	Clupea pallasii	Pacific herring	no
HERRWCVANI	Pacific herring W. Coast Van. Is.	Clupea pallasii	Pacific herring	no
LNOSESKA3CD	Longnose skate W. Coast Van. Is.	Raja rhina	Longnose skate	no
LNOSESKA4B	Longnose Skate Str. of Georgia	Raja rhina	Longnose skate	no
LNOSESKA5AB	Longnose Skate Queen Charlotte Sound	Raja rhina	Longnose skate	no
LNOSESKA5CDE	Longnose skate Hecate Strait	Raja rhina	Longnose skate	no
PANDALSMA14	Northern shrimp SMA 14	Pandalus borealis	Northern shrimp	no
PANDALSMA16	Northern shrimp SMA 16	Pandalus borealis	Northern shrimp	no
PANDALSMA18-19	Northern shrimp SMA 18-19	Pandalus borealis	Northern shrimp	no
PANDALSMAFR	Northern shrimp SMA FR	Pandalus borealis	Northern shrimp	no
PANDALSMAGTSE	Northern shrimp SMA GTSE	Pandalus borealis	Northern shrimp	no
PANDALSMAPRD	Northern shrimp SMA PRD	Pandalus borealis	Northern shrimp	no
PCOD5AB	Pacific cod Queen Charlotte Sound	Gadus macrocephalus	Pacific cod	no
PCODWCVANI	Pacific cod W. Coast Van. Is.	Gadus macrocephalus	Pacific cod	no
QROCKPCOASTIN	Quillback rockfish Pac. Coast (Inside)	Sebastes maliger	Quillback rockfish	no
QROCKPCOASTOUT	Quillback rockfish Pac. Coast (Outside)	Sebastes maliger	Quillback rockfish	no
SARDBC	Pacific sardine BC	Sardinops sagax	Pacific sardine	no
SSHRIMPSMAGTSE	Sidestripe shrimp SMA GTSE	Pandalopsis dispar	Sidestripe shrimp	no
SSHRIMPSMAPRD	Sidestripe shrimp SMA PRD	Pandalopsis dispar	Sidestripe shrimp	no
SSSHRIMPSMA14	Sidestripe shrimp SMA 14	Pandalopsis dispar	Sidestripe shrimp	no
SSSHRIMPSMA16	Sidestripe shrimp SMA 16	Pandalopsis dispar	Sidestripe shrimp	no
SSSHRIMPSMA18-19	Sidestripe shrimp SMA 18-19	Pandalopsis dispar	Sidestripe shrimp	no
SSSHRIMPSMAFR	Sidestripe shrimp SMA FR	Pandalopsis dispar	Sidestripe shrimp	no
YEYEROCKPCOASTIN	Yelloweye rockfish Pac. Coast (Inside)	Sebastes ruberrimus	Yelloweye rockfish	no

* "Assessed" refers to stocks assessed against an MSY reference point

Gapfilling: Due to high variance of annual catch estimates used to determine weighting of stock status scores within each region, estimates per stock and region were gapfilled by carrying back the mean of the first three available years, and carrying forward the mean of the last three available years. Gaps in stock assessment values were simply carried forward from the last observation.

Aquaculture

The Aquaculture (AQC) model compares the aquaculture harvest H within a region to its total harvest potential P, for both finfish (f) and bivalve (b) aquaculture (weighted by harvest of each aquaculture type).

$$X_{rgn,yr}^{AQC} = \frac{1}{H_f + H_b}\left(H_f \min(\frac{H_f}{P_{f,ref}},\ 1) + H_b \min(\frac{H_b}{P_{b,ref}},\ 1)\right)$$

Using aquaculture growth potential index data from Gentry et al. [9], we determined a reference harvest potential for finfish and bivalves for each region, in tonnes/km[2]. Designated aquaculture tenures [10] outline areas approved for aquaculture production, which we take to be a proxy for management targets. Multiplying the harvest potential by the area of designated aquaculture tenures for finfish and bivalves, we estimate the total sustainable harvest potential, in tonnes, for each region.

For both finfish and shellfish, a score of 100 reflects a harvest equal to the lower bound on potential calculated from (mean - 1 sd) of the growth potential index: $P_{ref} = P(\mu_{\Phi\prime} - \sigma_{\Phi\prime})$.

While harvest far above the estimated production potential may indicate unsustainable practices, particularly for high stocking densities of finfish, we did not apply an overproduction penalty due to the uncertainty inherent in production potential estimates and site-specific production methods.

Gapfilling: Because the time series of available data is short and shows high variance, we do not gapfill these layers, as assumptions are not likely to be valid. As such, this goal is scored only for 2011-2015.

Salmon

The Salmon sub-goal of Food Provision compares annual catch C for S = 13 indicator fisheries (S10 Table) to catch target C^{ref} for that year for that fishery, scoring 100 when the catch is between 60% and 100% of the catch target (the standard deviation of C/C_t across all stocks and years is 0.4, so 60% of catch target allows a 1 standard deviation buffer), dropping to a score of 25 as catch falls from 60% of target to zero, and dropping to 0 when the catch exceeds twice the target. Score is calculated separately for each unit and then all scores for all indicator stocks are averaged; a single score is applied equally across all OHIBC regions.

$$X_{SAL} = \begin{cases} 25 + 125C/C_t & \text{when } 0 \leq C/C_t \leq .60 \\ 100 & \text{when } .60 < C/C_t \leq 1.0 \\ 200 - 100C/C_t & \text{when } 1.0 < C/Ct \leq 2.0 \\ 0 & \text{else} \end{cases}$$

S10 Table: Salmon fisheries included in Wild-Capture Salmon and First Nations Resource Access Opportunities goals

species	fishery	FN Res. Access Opps (Escapements)	Wild-Capture Salmon (Catch)
Chinook	AABM North Coast	-	X
Chinook	AABM West Coast Vancouver Island	-	X
Chum	Fraser River	X	-
Chum	Johnstone Strait	-	X
Chum	southern	-	X
Coho	Inside F	X	X
Pink	Fraser River	X	X

S10 Table: Salmon fisheries included in Wild-Capture Salmon and First Nations Resource Access Opportunities goals

species	fishery	FN Res. Access Opps (Escapements)	Wild-Capture Salmon (Catch)
Sockeye	Fraser River Early Stuart	X	X
Sockeye	Fraser River Early Summer	X	X
Sockeye	Fraser River Late	X	X
Sockeye	Fraser River Summer	X	X
Sockeye	Skeena	X	-
Sockeye	Stikine - Non-Tahltan	X	-
Sockeye	Stikine - Tahltan	X	X
Sockeye	West Coast Vancouver Island	X	X

Gapfilling: none. For years prior to 2003, this subgoal is not scored.

First Nations Resource Access Opportunities

The First Nations Resource Access Opportunities (AO) goal examines access to four marine resources of broad FSC importance to First Nations communities across the British Columbia coast: wild-capture salmon, shellfish beds, herring spawn-on-kelp, and access to commercial fisheries.

The goal score for a given region and year is determined by an average of all available component scores for that region and year.

$$X_{rgn,yr}^{AO} = \frac{1}{c} \sum_{comp=1}^{c} X_{rgn,yr}^{comp}$$

The commercial fisheries, shellfish access, herring spawn access, and salmon access components are described below.

Commercial fisheries access

As a proxy for fisheries access, we compare the proportion of commercial fishing licenses allotted specifically for "aboriginal" license types or holders within each region by DFO [15] to the proportion of the region's population living in First Nations communities (based upon 2016 population of census subdistricts (csd) identified as First Nations communities (i.e., $csd = FN$), [17]). A score of 100 in this component indicates the proportion of FN-allocated licenses meets or exceeds the proportion of FN population, or 15%, whichever is greater.

$$X_{rgn,yr}^{AO,licenses} = \min\left(\frac{L_{rgn,yr}^{FN} / L_{rgn,yr}^{total}}{\max(Pop_{rgn}^{FN} / Pop_{rgn}^{total}, \quad 0.15)}, \quad 1 \right)$$

Fishing licenses allow access to specific Pacific Fisheries Management Areas (PFMAs). For each region and year, we count the number of licenses L (First Nations, and all) that allow access to each OHIBC region.

$$L_{rgn,yr}^{FN} = \sum_{PFMA=1}^{n} L_{PFMA}^{FN}$$

$$L_{rgn,yr}^{total} = \sum_{PFMA=1}^{n} L_{PFMA}$$

$$Pop_{rgn}^{FN} = \sum_{csd=1}^{m} (Pop_{csd} \times \mathbb{1}_{[csd=FN]})$$

$$Pop_{rgn}^{total} = \sum_{csd=1}^{m} Pop_{csd}$$

Gapfilling: none.

Shellfish harvest

To determine access to safe shellfish harvests, we determined the number of contamination-related shellfish closure days Cl in each fishery management subarea a for each year (data were available for 2009 to 2015) [14], and calculated an area-weighted mean number of closure-free days throughout each region. A score of 100 in this component indicates no closures due to contamination in the region.

$$X_{rgn,yr}^{AO,closures} = 1 - \frac{Cl_{rgn,yr}}{365}$$

$$Cl_{rgn,yr} = \frac{\sum_{a=1}^{n} Cl_a \times A_a}{\sum_{a=1}^{n} A_a}$$

Gapfilling: For years prior to 2009, component scores for each region for 2009 were carried backward; for 2016, the 2015 region-component scores were used.

Herring spawn abundance

Herring spawn index data [16] estimate the mean density of herring spawn available in each herring section. We aggregate these to OHIBC region, applying a rolling three-year mean to smooth typical interannual variations to calculate herring spawn abundance H for each region for the years 1940-2016. A score of 100 in this component indicates a smoothed herring spawn index value H that meets or exceeds the reference value H_{ref} as the mean value within each region across a 20-year reference period from 1940-1960. The reference period was selected to estimate historic abundance prior to a crash in herring stocks in the 1960s.

$$X_{rgn,yr}^{AO,herring\ spawn} = \frac{H_{rgn,yr}^{smoothed}}{H_{rgn,ref}}$$

$$H_{rgn,yr}^{raw} = \sum_{section=1}^{n} H_{section,yr}$$

$$H_{rgn,yr}^{smoothed} = \frac{1}{3}(H_{rgn,yr-2}^{raw} + H_{rgn,yr-1}^{raw} + H_{rgn,yr}^{raw})$$

Gapfilling: none.

Wild caught salmon

Wild salmon escapements near a defined escapement targets ensure access to healthy salmon stocks in the future. We calculate the ratio of annual escapement against escapement targets for twelve indicator salmon stocks across four species (chum, coho, pink, and sockeye) [11–13] (See Table S10 for a list of stocks included in this component). For each stock, score increases linearly from 0 when that stock's escapement to target ratio is at or below 0.4 (approximately one standard deviation below target) to 1 when the ratio is at or above 1.0. All stock scores are averaged into a single salmon score that is applied equally for all regions.

$$X_{yr}^{AO,salmon} = \frac{1}{n}\sum_{stock=1}^{n} E'_{stock,yr}$$

for all regions, where

$$E'_{stock,yr} = \begin{cases} 0 & \text{when } \frac{E_{stock,yr}}{E_{stock,target}} < 0.4 \\ 1 & \text{when } \frac{E_{stock,yr}}{E_{stock,target}} \geq 1 \\ \frac{E_{stock,yr} - 0.4E_{stock,target}}{0.6E_{stock,target}} & \text{otherwise} \end{cases}$$

Gapfilling: none. For years prior to 2003, this component does not contribute to scores.

Livelihoods

Coastal Livelihoods are scored as the average of First Nations Livelihoods (LVF) and Non-First Nations Livelihoods (LVN) subgoals.

$$X_{rgn,yr}^{LV} = \frac{1}{2}\left(X_{rgn,yr}^{LVF} + X_{rgn,yr}^{LVN}\right)$$

The LVF and LVN subgoals are described below. For both subgoals, gapfilling of median income, employment, and population for each census subdistrict were all based on linear interpolation between census years 1996, 2001, 2006, 2011, and 2016.

First Nations Livelihoods

The First Nations Livelihoods model is based on job and wage data for coastal First Nation communities. Employment data by industry within British Columbia was not sufficiently detailed to identify jobs and wages for marine-dependent sectors. Instead we use a population-weighted average of employment rates (E = 1 - unemployment rate U) [17] and inflation-adjusted median wage W

[17] within the First Nation-specific census subdistricts $csd \in \{\text{FN}\}$ [17] that fall within OHIBC inland boundaries.

$$X_{rgn,yr}^{LVF} = \frac{1}{2}\left(\frac{E_{rgn,yr}^{FN}}{E_{rgn,yr}^{ref}} + \frac{W_{rgn,yr}^{FN}}{W^{ref}}\right)$$

Because no objectively defined reference point for employment rate was available, we calculate a reference point as a relative value on a moving baseline: the value in the current year relative to the mean value in a moving 5-year reference period, starting 5 years prior to the current year. To enable comparison between First Nations and non-First Nations employment rate, we use the higher of the two rolling means as the reference point for both. This reflects an implicit goal of maintaining coastal livelihoods and economies on short time scales, allowing for decadal or generational shifts in what people want and expect for coastal livelihoods and economy.

$$E_{rgn,yr=t}^{ref} = \max\left(mean(E_{rgn,yr\in[t-5,t-1]}^{FN}),\ mean(E_{rgn,yr\in[t-5,t-1]}^{non-FN}) \right)$$

We defined the wage reference point as the highest observed inflation-adjusted wage across all OHIBC regions and all years for both First Nation and non-First Nation communities.

$$W^{ref} = \max(W^{FN}, W^{non-FN}) \text{ across all regions and years}$$

Employment and wage information are reported at the census subdistrict level, and a population-weighted mean value for each region is calculated based upon census subdistricts identified as First Nations.

$$E_{rgn,yr}^{FN} = \frac{\sum_{csd\in FN} E_{csd} \times pop_{csd}}{\sum_{csd\in FN} pop_{csd}}$$

$$W_{rgn,yr}^{FN} = \frac{\sum_{csd\in FN} W_{csd} \times pop_{csd}}{\sum_{csd\in FN} pop_{csd}}$$

Non-First Nations Livelihoods

The non-First Nations Livelihoods model is identical to the First Nations Livelihoods model, except that employment and wage data are based on non-First Nation communities, identified as non-First Nation-specific census subdistricts $csd \in \{\text{non-FN}\}$ [17] that fall within OHIBC inland boundaries.

$$X_{rgn,yr}^{LVN} = \frac{1}{2}\left(\frac{E_{rgn,yr}^{non-FN}}{E_{rgn,yr}^{ref}} + \frac{W_{rgn,yr}^{non-FN}}{W^{ref}}\right)$$

$E_{rgn,yr=t}^{ref}$ and W^{ref} are identical to those used in the LVF subgoal.

Employment and wage information are reported at the census subdistrict level, and a population-weighted mean value for each region is calculated based upon census subdistricts *not* identified as First Nations.

$$E_{rgn,yr}^{non-FN} = \frac{\sum_{csd \notin FN} E_{csd} \times pop_{csd}}{\sum_{csd \notin FN} p\ op_{csd}}$$

$$W_{rgn,yr}^{non-FN} = \frac{\sum_{csd \notin FN} W_{csd} \times pop_{csd}}{\sum_{csd \notin FN} p\ op_{csd}}$$

Tourism and Recreation

For this goal, we use number of visitors N to coastal parks [18] and visitor centers [19] within a defined coastal region as a measure of tourist activity. Our area of "coastal interest" is defined by a buffer extending 15 km inland from the coastline. Visitors in a given year are compared to a moving reference point of mean visitation over the prior five year period. Park visits and visitor center visits are scored separately then averaged. Regions with no park or visitor center data were given NA scores.

$$X_{rgn,yr}^{TR} = \frac{1}{2}\left(X_{rgn,yr}^{park} + X_{rgn,yr}^{vis.ctr}\right)$$

where

$$X_{rgn,yr=t}^{park} = \frac{N_{rgn,yr=t}^{park}}{0.2 \sum_{yr=t-5}^{t-1} N_{rgn,yr}^{park}}$$

and similar for visitor center visits.

Gapfilling: Due to the "no net loss" reference point, we chose to not backfill values prior to 2007. Therefore this goal was not scored prior to 2007.

Sense of Place

Sense of Place score is the average of Lasting Special Places and Iconic Species subgoals.

$$X_{rgn,yr}^{SP} = \frac{1}{2}\left(X_{rgn,yr}^{LSP} + X_{rgn,yr}^{ICO}\right)$$

The Iconic Species and Lasting Special Places subgoals are described below.

Iconic Species

The Iconic Species model measures the percentage of iconic species in each extinction risk category. Species list is based upon input from Karin Bodtker and Andrew Day of the Vancouver Aquarium/CORI project. A full list of included iconic species can be found in S11 Table. Threat weights were assigned based on the COSEWIC province-level threat status where available [23] (covering most species on the list) and IUCN threat status [20] (for species without an available COSEWIC assessment).

In addition to including only a subset of species in the Species goal, the Iconic Species subgoal is not based on area-weighted average of species within a region, only on whether a species is present within a region. A score of 100 indicates all iconic species are at "Least Concern" status.

$$X_{rgn,yr}^{ICO} = \frac{1}{n} \sum_{spp=1}^{n} S\,tatus_{rgn,yr}^{spp}$$

where $Status_{spp,yr}$ score corresponds to IUCN extinction risk categories: "Least Concern" = 1.0, "Near Threatened" = 0.8, "Vulnerable" = 0.6, "Endangered" = 0.4, "Critically Endangered" = 0.2.

S11 Table: Species included in OHIBC Iconic Species

**Scientific name**	**Common name**
Ammodytes hexapterus	Pacific Sand Lance
Ardea herodias	Great Blue Heron
Balaenoptera acutorostrata	Minke Whale
Balaenoptera borealis	Sei Whale
Balaenoptera edeni	Bryde's Whale
Balaenoptera musculus	Blue Whale
Balaenoptera physalus	Fin Whale
Brachyramphus marmoratus	Marbled Murrelet
Branta canadensis	Canada Goose
Cetorhinus maximus	Basking Shark
Clupea pallasii	Pacific Herring
Delphinus delphis	Common Dolphin
Dermochelys coriacea	Leatherback Turtle
Enhydra lutris	Sea Otter
Eschrichtius robustus	Gray Whale
Eubalaena japonica	North Pacific Right Whale
Eumetopias jubatus	Steller Sea Lion
Gavia immer	Common Loon
Grampus griseus	Risso's Dolphin
Haliaeetus leucocephalus	Bald Eagle
Haliotis kamtschatkana	Northern Abalone
Hippoglossus stenolepis	Halibut
Hypomesus pretiosus	Surf Smelt
Lagenorhynchus obliquidens	White-Sided Dolphin
Lamna ditropis	Salmon Shark
Megaptera novaeangliae	Humpback Whale
Mesoplodon densirostris	Blainville's Beaked Whale
Oncorhynchus gorbuscha	Pink Salmon
Oncorhynchus kisutch	Coho Salmon
Oncorhynchus nerka	Sockeye Salmon
Oncorhynchus tshawytscha	Chinook Salmon
Phoca vitulina	Harbor Seal
Phocoena phocoena	Harbor Porpoise

S11 Table: Species included in OHIBC Iconic Species

Scientific name	Common name
Phocoenoides dalli	Dall's Porpoise
Physeter macrocephalus	Sperm Whale
Pseudorca crassidens	False Killer Whale
Ptychoramphus aleuticus	Cassin's Auklet
Squalus suckleyi	Spiny Dogfish
Thaleichthys pacificus	Eulachon
Zalophus californianus	California Sea Lion
Ziphius cavirostris	Cuvier's Beaked Whale
Orcinus orca pop. 2	Killer Whale (NE Pac. Offshore)
Orcinus orca pop. 3	Killer Whale (West Coast Transient)
Orcinus orca pop. 5	Killer Whale (NE Pac. Southern Resident)
Orcinus orca pop. 6	Killer Whale (NE Pac. Northern Resident)

Gapfilling: Conservation status for each species is based on a last observation carried forward, i.e., the current status is based on the most recent prior assessment. For years prior to the first assessment, the first assessment status is carried backward.

Lasting Special Places

Lasting Special Places measures the percentage of protected coastal marine and coastline area in each region, against a 30% reference target [44]. We include protected areas within coastal waters (MPAs, within 3nmi of shore) for marine special places assuming that sense of place is limited to areas readily accessible to or visible from the shoreline. For land-based protected areas (PAs), we include coastal sub-watersheds as they are intrinsically connected to the marine system. To determine protection status using a variety of sources, including the World Database of Protected Areas [24], British Columbia parks and protected areas, and tribal parks. While MaPP Special Management Zones communicate areas of deep historical, traditional, and cultural importance, they are not yet formally protected, so these regions were excluded from the analysis.

$$X_{rgn,yr}^{LSP} = \frac{1}{2}\left(\min\left(\frac{A_{rgn,yr}^{MPA}}{0.30A_{rgn,marine}},\ 1\right) + \min\left(\frac{A_{rgn,yr}^{PA}}{0.30A_{rgn,coastal}},\ 1\right)\right)$$

Gapfilling: none.

Biodiversity

Biodiversity status averages the condition of species (Species subgoal) and biodiversity-supporting biogenic habitats (Habitats subgoal).

$$X_{rgn,yr}^{BD} = \frac{1}{2}\left(X_{rgn,yr}^{SPP} + X_{rgn,yr}^{HAB}\right)$$

The Species and Habitats subgoals are described below.

Species

The Species model measures the average threat status, defined by COSEWIC province-level threat assessments [23] where available and IUCN Red List threat assessments [20] elsewhere, of all species found in each region, weighted by each species' area of distribution A within the region. See S12 Table for a count of species by taxa included in this assessment. Note that the species included in the Iconic Species subgoal are also represented here, making up approximately 9% of the species included in this subgoal.

S12 Table: Taxonomic groups included in OHIBC Species

phylum	class	n
Arthropoda	Malacostraca	4
Chordata	Actinopterygii	218
Chordata	Aves	134
Chordata	Cephalaspidomorphi	3
Chordata	Chondrichthyes	35
Chordata	Mammalia	36
Chordata	Myxini	2
Chordata	Reptilia	4
Echinodermata	Holothuroidea	6
Mollusca	Bivalvia	1
Mollusca	Cephalopoda	23
Mollusca	Gastropoda	1
Tracheophyta	Liliopsida	6

The upper reference point for the Species sub-goal is to have all species at a risk status of Least Concern. As in OHI global assessments, we scale the lower end of the goal to be 0 when 75% of species are extinct, a level comparable to the five documented mass extinctions that would constitute a catastrophic loss of biodiversity.

$$X_{rgn,yr}^{SPP} = \max\left(\frac{\text{mean}(Status_{rgn,yr}) - 0.25}{0.75}, 0\right)$$

$$\text{mean}(Status_{rgn,yr}) = \frac{\sum_{spp=1}^{n} A_{spp,rgn} \times Status_{spp,yr}}{\sum_{spp=1}^{n} A_{spp,rgn}}$$

where $Status_{spp,yr}$ score corresponds to IUCN extinction risk categories: "Least Concern" = 1.0, "Near Threatened" = 0.8, "Vulnerable" = 0.6, "Endangered" = 0.4, "Critically Endangered" = 0.2.

Gapfilling: Conservation status for each species is based on a last observation carried forward, i.e., the current status is based on the most recent prior assessment, rather than a linear interpolation. For years prior to the first assessment, the first assessment status is carried backward.

Habitats

Habitats score is the mean condition of each biodiversity-supporting habitat for each region and year. Habitats included in the assessment are soft-bottom benthic habitats [2], salt marsh [1], and ecologically/biologically significant areas (EBSAs). EBSAs are determined by the DFO as areas with oceanographic, physical, or ecological conditions with special significance [27]; for our purposes we include only EBSAs related to biodiversity-supporting structure, including sponge reefs, deep water corals, hydrothermal vents, and seamounts.

$$X_{rgn,yr}^{HAB} = \frac{1}{n_{rgn,yr}^{hab}} \sum_{hab \in sm,sb,ebsa} X_{rgn,yr}^{hab}$$

The Habitats sub-goal assess the health condition of each habitat present in a region, based upon external trawling pressures (for soft-bottom habitat and EBSAs) and coverage area relative to historical baseline (for saltmarsh habitats).

Subtidal soft bottom habitat health is the inverse of average trawl effort across the region (i.e. hours of trawl per km^2) on soft-bottom habitat areas [2], relative to a reference point of the maximum trawl effort E_ref observed in any 4 km x 4 km cell for any year in the dataset [6].

$$X_{rgn,yr}^{sb} = \frac{1}{n} \sum_{cell=1}^{n} E_{trawl} / E_{ref}$$

EBSA health is the inverse of the average trawl presence in a given year (i.e. trawled area relative to total EBSA area) on EBSA-associated areas [27]. Note this is not effort-based as for soft-bottom habitats, since these slow-growing structures are far slower to recover than soft bottom sediment.

$$X_{rgn,yr}^{ebsa} = \frac{1}{n} \sum_{cell=1}^{n} \mathbb{1}_{trawl=TRUE}$$

Saltmarsh condition is calculated as the extent of a region's saltmarshes within 1 km of the shoreline, as noted by a 30 m resolution land use raster [1]. The reference point is the saltmarsh extent according to the 1990 land use raster.

$$X_{rgn,yr}^{sm} = A_{rgn,yr}^{sm} / A_{rgn,1990}^{sm}$$

Gapfilling: EBSA and soft bottom habitat pressures values prior to 2005 were gapfilled using next observation carried forward, while 2016 values were carried forward from 2015. Saltmarsh condition was gapfilled in the same manner as described for Coastal Protection subgoal.

Clean Waters

The Clean Waters goal score is calculated as the geometric mean of its four components: eutrophication (nutrients), chemicals, pathogens and marine debris. Each component layer estimates the pressure prs_{comp} due to that component on the system, so each component score is calculated as $1 - prs_{comp}$.

$$X_{rgn,yr}^{CW} = \prod_{comp=1}^{c} (1 - prs_{comp,yr})^{1/c}$$

The chemical, nutrient, pathogen, and marine debris components are described below.

Chemical pollution

Chemical pollution was measured as the average of land-based organic and inorganic pollution from agricultural pesticide use and runoff from impervious surfaces, respectively, and ocean-based pollution from commercial shipping and ports [45]. Organics are based on rasters of modeled plumes at 934 m resolution and are available for 2002-2013 [28]; these rasters are masked to the OHIBC study region, log transformed (log$(x+1)$), and rescaled from 0 to 1 where 1 indicates the 99.99%ile of the log-transformed values. Inorganics and ocean-based pollution are similar, though the layers are for a single year based on Halpern et al. [45]. These layers are already log-transformed to a global reference point; here they are masked to the OHIBC study region and rescaled where 1 indicates the highest observed value in the OHIBC study region. The chemical pressure score for each region is the mean chemical pressure score of all cells within the region.

$$prs_{cell,yr}^{chem} = \min([prs_{cell,yr}^{chem,organic} + prs_{cell}^{chem,inorganic} + prs_{cell}^{chem,ocean}],1)$$

$$prs_{rgn,yr}^{chem} = \text{mean}(prs_{cell \in rgn,yr}^{chem})$$

Gapfilling: Region component values for 2002 were carried back to the start of the time series; values after 2013 were carried forward to the end.

Nutrient pollution

Modeled land-based nitrogen input for 2002-2013 [28] was used as a proxy for nutrient input. As for organic chemical pollution, it was masked to the OHIBC study region, transformed as log$(x+1)$, and rescaled 0 to 1 based on the 99.99%ile of values.

$$prs_{rgn,yr}^{nutr} = \text{mean}(prs_{cell \in rgn,yr}^{nutr})$$

Gapfilling: Region component values for 2002 were carried back to the start of the time series; values after 2013 were carried forward to the end.

Pathogens

Due to a lack of information on direct measurements of human pathogens in coastal waters, we used a proxy measure for pathogens: the population density of inland regions with unimproved wastewater treatment (i.e. population density on septic, storage-and-haulage, or no treatment) relative to the highest population density of any OHIBC region.

At risk densities were based on Municipal Water Use Report surveys from 2004, 2006, and 2009 [30–32]. These reports estimate the percent of population served by sewers, private septic systems, and sewage hauling, based on municipality size. We defined "at risk" as population not on sewer systems. For First Nations communities, we relied upon the National Assessment of First Nations Water and Wastewater Systems [29]. A digitized map of inspected wastewater systems classified as

high, medium, and low risk was used to estimate the average risk for First Nations communities (as determined by census subdivision) within each OHIBC region.

$$\rho_{rgn,yr}^{at-risk} = \frac{\sum_{muni \in rgn} p\,ct_{muni,yr}^{at-risk} \times pop_{muni,yr}}{A_{rgn}}$$

$$\rho_{max,yr} = \max_{rgn}\left(\frac{\sum_{muni \in rgn} p\,op_{muni,yr}}{A_{rgn}}\right)$$

$$prs_{rgn,yr}^{patho} = \frac{\rho_{rgn,yr}^{at-risk}}{\rho_{max,yr}}$$

Gapfilling: none.

Marine debris

The status of marine debris was estimated using modeled mass density of marine plastics (in kg/km^2) from Van Sebille et al. [33] on a 1° global grid. We interpolated using a thin-plate spline method to extend this grid into the Strait of Georgia and coastal fjords, then reprojected to BC Albers projection at 1000 m resolution, masked to the OHIBC region of study. The data were then rescaled from 0 to 1 based on the highest value found within the OHIBC study region. There is no time series for this layer.

$$prs_{rgn}^{debris} = \text{mean}(prs_{cell \in rgn}^{debris})$$

Gapfilling: As there is no time series for this component, all years were scored the same.

Supporting Methods: Trend

Trend T represents the proportional change in status X over a recent past period, and is used to infer likely changes in status in the near future. For most goal models (except SPP and ICO, noted below), trend is calculated as the slope estimate of a linear regression of status for the prior five-year period, divided by the status in the earliest year of the five-year period; this result is multiplied by five to indicate the likely change in status over the next five years.

$$T_{yr=t} = 5 \times \frac{(dX/dt)_{yr \in t-4:t}}{X_{yr=t-4}}$$

In general, trend is constrained to a range of +1 to -1. If a goal status reaches 100, trend is limited to non-positive values; similarly, if a goal status reaches zero, trend is limited to non-negative values.

For the Species (SPP) and Iconic Species (ICO) subgoals, we converted IUCN species-specific trend information (e.g., “increasing”, “decreasing”, “stable”) to numeric values, based on a regression of species status (only for species whose status has been assessed multiple times) against these categories. For ICO, trend is the average species-specific trend of all species found within a region; for SPP, trend is the area-weighted average of these species specific trends.

Supporting Methods: Pressures

The pressure score, p, describes the cumulative impact of ecological and social stressors in a given year and region which tend to depress the goal score in future years. Pressure scores range from 0 to 1, and include both ecological (p_E) and social pressures (p_S), such that:

$$p = \gamma p_E + (1 - \gamma) p_S$$

where $\gamma = 0.5$ is the relative weight for ecological vs. social pressures categories. We default to equal weighting as little evidence was available to justify or quantify unequal weights between ecological and social pressures categories. It may be that future work can inform unequal weighting terms γ for individual goals.

For each goal, subgoal, or goal element (e.g. specific habitat), we calculated pressures as an impact-weighted cumulative impact for each pressure category p_{ecol}^{goal} and p_{soc}^{goal}. Impact weights are based on a goal's sensitivity $w_{s_i}^{goal}$ to specific stressors s_i ranked as low ($w_{s_i}^{goal} = 1$), medium ($w_{s_i}^{goal} = 2$), high ($w_{s_i}^{goal} = 3$), or no impact ($w_{s_i}^{goal} = NA$), as determined by peer-reviewed literature and expert judgment (S6 Fig. shows the matrix of stressors, goals, and weights). The denominator represents the maximum stressor impact weight for that category and goal. If *cumulative* pressure load for a goal/component combination exceeds the maximum possible stressor intensity, we cap it to 1.0, i.e. the equivalent to an individual stressor at maximum stress and intensity.

$$p_{cat}^{goal} = \min\left(\frac{1}{w_{cat,max}^{goal}} \sum_{i=1}^{N} w_{s_i}^{goal} \times s_i, \quad 1\right)$$

		po_chemical	po_chemical_3nm	po_pathogen	po_nutrient	po_nutrient_3nm	po_trash	sp_alien	sp_genetic	aq_mammals	aq_incidental	aq_benthic	hd_subtidal_sb	hd_intertidal	hd_logging	fp_fis_discards	fp_fis_landings	cc_sst	cc_acid	cc_uv	cc_slr	ss_cwbi_all	ss_cwbi_fn
		ecological																				social	
goal	element	pollution						aq_alien_species					habitat_destr			fishing_prs		climate_change				social	
First Nations Res. Access Opp.	shellfish_closures		2	1		3						1	1	1	1			2	3		1		1
	salmon	1			1			2	2						2	1	2	1					1
	fn_licenses	1			1			1	1		1	1	2	1	1	1	3	1					1
	herring_spawn		2			3		1			1		2	2	1		3	2			1		1
Coastal Protection	coastal_forest													1	2						1	1	
	saltmarsh		1			2		1						3	2						3	1	
Carbon Storage	coastal_forest													1	2						1	1	
	saltmarsh		1			2		1						3	2						2	1	
Clean Waters			3	3		3	3								1							1	
Wild-Capture Fisheries		1			1			1	1		1	1	2	1	1	1	3	1				1	
Habitats	saltmarsh		1			2		1						3	2						2	1	
	soft_bottom	2			2			1				2	3		1	1	3		1			1	
	ebsa	2			2			1				1	3		1	1	3	1	1			1	
Iconic Species		1			1		2	1	1	3			1	1	1	1	2	1	1	1		1	
First Nations Livelihoods																							3
Non-First Nations Livelihoods																						3	
Lasting Special Places			2			2	3	1				1		3	1						1	1	
Mariculture			2	1		3		1				2			1			2	3		1	1	
Wild-Capture Salmon		1			1			2	2						2	1	2	1				1	
Species		2			3		1	1	1	1	1	1	3	2	1	1	3	1	1	1	1	1	
Tourism & Recreation			3	3		3	3			1				1	1						2	1	

S6 Fig.: Pressures matrix. Stressor layers (horizontal axis) are grouped into ecological and social pressures categories. The impact (pressure) of each stressor layer acting on a

particular goal or component (vertical axis) is weighted from 1 to 3. Blank cells indicate a stressor has negligible impact on a goal.

Ecological pressure

We included five subcategories of ecological stressors relevant to British Columbia: fishing pressure, habitat destruction, climate change, water pollution, and species introductions (invasive species and genetic escapes). Each pressure category may include several stressors in individual layers. The intensity of each stressor within each OHI region is scaled from 0 to 1, with 1 indicating the highest stress relative to a defined reference point, often the highest observed stress within the OHIBC study area.

The overall ecological pressure, p_E, acting on each goal for each region and year was calculated as the weighted average of the pressure scores, p, for each subcategory, i, acting on that goal, with weights set as the maximum rank in each pressure category ($w_{i,max}$) for each goal, such that:

$$p_E^{goal} = \frac{\sum_{cat=1}^{N}(w_{cat,max}^{goal} \times p_{cat}^{goal})}{\sum_{cat=1}^{N} w_{cat,max}^{goal}}$$

Stressors that have no impact (i.e. $w_{s_i}^{goal} = NA$) drop out of the calculations and do not affect the pressures score.

A note on ecological pressures not included in this assessment: A number of likely significant pressures on BC's coastal ecosystems were not able to be included in this assessment. For example, we were unable to include impacts of terrestrial mining or log boom presence due to lack of data availability at a usable spatial and time series resolution. In future assessments, additional stressors can easily be incorporated into the pressures matrix as new data become available, though consideration should also be given to potential resilience measures that might ameliorate the impacts of those additional stressors.

Social pressures

Social pressures describe the lack of effectiveness of government and social institutions. Social stressors are described for each region and year on a scale of 0 to 1 (with one indicating the highest pressure).

The Community Well Being (CWB) Index [39] produced by Indigenous and North Affairs Canada combines indicators including education, labour force activity, income and housing to provide insights into the social well being of Indigenous and non-Indigenous communities in Canada. We calculate social pressures as the population-weighted average of community-level CWB scores, subtracted from 1 to indicate that low community well being indicates ineffective governance and social institutions.

$$p_{CWB} = 1 - \frac{\sum_{i=1}^{N}(CWB_{csd} \times pop_{csd})}{\sum_{i=1}^{N} pop_{csd}}$$

This component is calculated separately for all communities in each OHIBC region, applied to goals describing benefits to all BC residents, and for First Nation communities specifically (as noted by census subdistrict designation), applied to goals only applicable to First Nation communities. Maximum pressure ($p_{CWB} = 1$) occurs when all CWB indicators are at 0 out of 100, while minimum pressure ($p_{CWB} = 0$) occurs when all indicators are at 100.

The CWB is also used as an indicator of social resilience, as described below.

A note on the assumption of linear and additive response to pressures: As in the global OHI pressures model, we assume for this OHIBC assessment that is that each goal responds to changes in intensity of ecological stressors in a linear and additive fashion. Such an assumption obviously fails to capture likely non-linear responses and synergistic or antagonistic interactions among stressors, but such responses remain poorly characterized so we could not justify including such responses in our model.

Supporting Methods: Resilience

Resilience for each goal and region, r, is based on three components: ecological integrity, r_{ecol}; regulatory efforts that target specific ecological pressures, r_{reg}; and social integrity, r_{soc}. The r_{ecol} and r_{reg} combine to address resilience to ecological pressures, while r_{soc} addresses social pressures. Each resilience category contains one or more layers reflecting the magnitude of resilience within each region for each year; layers are "activated" to address specific pressures acting on specific goals based on a resilience matrix (S7 Fig.), and active layers are summed to determine a score for each resilience category. Each layer is constrained from 0 to 1.

$$r = \gamma(\frac{r_{ecol} + r_{reg}}{2}) + (1 - \gamma)r_{soc}$$

These components are weighted such that resilience to ecological pressures (i.e., $r_{ecol} + r_{reg}$) and resilience to social pressures (i.e., r_{soc}) reflect the proportional contribution of ecological and social pressures in the pressures model, i.e. $\gamma = 0.5$.

		species_diversity_eez	species_diversity_3nm	aq_regulation	fp_mpa_coast	fp_mpa_eez	fp_biomass_removal	hd_trawl_reduction	hd_mpa_coast	hd_mpa_eez	mapp_resilience	cwbi_all	cwbi_fn
		ecosystem		regulatory							social		
**goal**	**element**	ecological		aq_spp	fishing_pressure			habitat_destruction			social		
First Nations Res. Access Opp.	shellfish_closures		x	x	x		x		x		x		x
	salmon		x	x	x		x		x		x		x
	fn_licenses		x		x		x		x		x		x
	herring_spawn		x	x	x		x		x		x		x
Coastal Protection	coastal_forest								x		x	x	
	saltmarsh			x					x		x	x	
Carbon Storage	coastal_forest								x		x	x	
	saltmarsh			x					x		x	x	
Clean Waters				x							x	x	
Wild-Capture Fisheries		x				x	x			x	x	x	
Habitats	saltmarsh		x	x					x		x	x	
	ebsa	x		x		x	x	x		x	x	x	
	soft_bottom	x		x		x	x			x	x	x	
Iconic Species		x		x		x	x			x	x	x	
Species				x		x	x			x	x	x	
First Nations Livelihoods											x		x
Non-First Nations Livelihoods											x	x	
Lasting Special Places											x	x	
Mariculture				x							x	x	
Wild-Capture Salmon		x		x		x	x			x	x	x	
Tourism & Recreation											x	x	

S7 Fig.: Resilience matrix. Resilience layers are grouped into regulatory, ecological, and social resilience. Regulatory resilience is further divided into categories that align with ecological pressure categories. For each goal, relevant resilience layers mitigate the effects of the pressures acting upon that goal.

Ecological integrity

An intact biodiverse ecosystem provides general resilience to ecological pressures by ensuring the system's ability to maintain functionality in the face of stressors imposed by human activity and climate change. For OHIBC, we consider the area-weighted average conservation status of all species found in the coastal zone (3 nmi offshore) (as resilience to coastal pressures) and found within the entire EEZ (as resilience to pressures not limited to the coast). The area-weighted average conservation status is calculated in the same manner as the Species subgoal.

Regulatory resilience

Regulatory resilience describes the institutional structures, rules, and regulations that directly address ecological pressures from human interactions with the marine system. For OHIBC we examined regulatory resilience to address three categories of pressure that correspond with : fisheries/biomass removal, habitat destruction, and aquaculture.

Where possible, we scored regulatory resilience based on a combination of a) existence of meaningful regulation, b) enforcement of regulation, and c) compliance with regulation.

Aquaculture regulatory resilience

Aquaculture regulatory data [34,40] are not spatialized to the OHIBC region level, so scores are calculated across the entire BC EEZ and applied equally to all regions.

- Presence: The existence of these data implies existence of regulation; score of 1 across all years.
- Enforcement: This is based on frequency of audits relative to some reference point.
  - For enforcement we combine scores for (fish health audits)/(active facilities), (sea lice audits)/(active facilities) and (benthic surveys)/(active facilities) using a geometric mean: enforcement across all facets must be high to achieve a strong resilience score. Poor enforcement on any facet indicates weakness in regulatory enforcement.
  - For fish health and benthic surveys, the reference point is the max seen across all years for each metric. Score is $\frac{\text{\% sites audited}}{(\text{\% sites audited})_{max}}$.
  - For sea lice, the reference point is 50%, per DFO policy. Score is $\frac{\text{\% sites audited}}{50\%}$.
- Compliance: Here we use two compliance metrics, reporting and violations. These are averaged.
  - Violations/Site where reference point is max seen across the data. Score is $1 - \frac{violations/site}{(violations/site)_{ref}}$.
  - Reporting Compliance comparing non-compliant reports to total reports, where reference value is 100% of reports marked non-compliant. Score is $1 - \frac{\text{noncompliant reports}}{\text{total reports}}$.

Overall resilience score is calculated as:

$$r_{AQ} = \frac{\text{reg presence} + \text{reg enforcement} + \text{reg compliance}}{3}$$

Marine Protected Area regulatory resilience

Marine protected areas shield biodiversity from pressures due to fisheries exploitation and habitat destructive practices. Data on MPAs comes from UNEP WDPA [24], BC Province Parks and Ecologically Protected Areas, and tribal parks. Reference point for MPA coverage is 30% of marine area [44]. Specific enforcement and compliance data are not readily available, so we use existence of a management plan for each MPA [42] as a proxy for management effectiveness; ideally, all MPAs would be subject to a published management plan.

MPA resilience is calculated at two scales to account for pressures that act at different scales: system-wide pressures (entire EEZ) and coastal pressures (the 3 nmi coastal zone).

$$r_{MPA} = \frac{A_{MPA}}{0.30A_{region}} \times \frac{\text{MPAs with management plan}}{\text{MPAs total}}$$

Fishing management regulatory resilience

Fishing regulations increase ecological resilience by limiting unintended biomass removal. Region-specific data were not available, so we calculated scores for the overall BC EEZ and applied scores equally to all regions.

• Presence: For all years of the study, the Fisheries Act has been in place; therefore $reg.presence = 1$ for all years.
• Enforcement: We use as a metric of enforcement the number of fisheries officers per fishing vessel $N_{officers}/N_{vessels}$ for each year; as a reference point as the maximum observed officers per vessel for any year. License data was supplied by DFO [15] and fisheries officer count is based on groundfish enforcement [41].
• Compliance: As a metric of compliance, we use observer coverage in groundfish fishery [41]. For all years, observer coverage is reported as 100%, so $reg.compliance = 1$ for all years.

$$r_{FP} = \frac{\text{reg presence} + \text{reg effectiveness} + \text{reg compliance}}{3}$$

Social resilience

Social resilience describes the social integrity of coastal communities that allow for adaptive responses to social and ecological pressures. We calculate social resilience scores by region separately for First Nation communities only (for First Nations-specific goals and subgoals) and for all communities (for all other goals and subgoals).

Community Well Being Index

The Community Well Being Index (CWB) [39] informs both our social pressures (as low scores indicate lack of effective social institutions) and our social resilience (conversely, high scores indicate functional social structures).

$$r_{CWB} = \frac{\sum_{i=1}^{N}(CWB_{csd} \times pop_{csd})}{\sum_{i=1}^{N} p\,op_{csd}} = 1 - p_{CWB}$$

As for pressures, this component is calculated separately for First Nation communities only and for all communities in each OHIBC region.

MaPP

The Marine Plan Partnership involved eliciting input and advice from member First Nations and BC Province experts to develop marine plans based on the best available science and local and traditional knowledge. MaPP resilience accounts for the adaptive benefits of engaging in the planning process beginning in 2011, as well as a presumption of improved compliance and self-monitoring once the plans were announced in 2015.

$$r_{MaPP,rgn} = \mathbb{1}_{rgn \in MaPP} \times \frac{process + enforcement + compliance}{3}$$

Supporting Methods: Data Selection Criteria

OHIBC incorporates 76 layers, constructed from dozens of datasets across social, economic, and environmental domains, to calculate status, pressures, and resilience for each goal. Ideally, every dataset would be an excellent "fit" for the needs of the calculation. In addition, each dataset would

ideally provide the spatial and temporal resolution to allow OHIBC scores to distinguish differences in each goal among regions and from year to year, and the spatial and temporal extent to adequately assess the entire region across the entire study period. We ranked each OHIBC dataset across three dimensions to identify strengths and weaknesses, and to highlight data gaps where effort and resources could increase the utility of a dataset to this OHIBC assessment. Note that these rankings are based on criteria specific to OHIBC, and may not reflect the utility of the dataset to an assessment at a different scale.

Methods

We identified three dimensions of data that affect the ability to calculate meaningful goal scores and one dimension that pertains to OHI's open science philosophy. For each of these dimensions, a dataset was scored 0.0, 0.5, or 1.0 on multiple facets as applicable (S13 Table). The dimensions and methods are loosely based upon methods described in Fritz *et. al.* [46].

- Spatial dimension: OHIBC aims to identify differences and patterns in goal status, pressures, and resilience across the seven regions included in the assessment.
    - Spatial extent: Ideally, spatial data encompass the entire area of interest, i.e., Canada's Pacific EEZ out to the shelf break. Scored as 1.0 if the dataset includes data across the entire study area of interest; 0.5 if the dataset includes most but not all OHIBC regions (e.g., MaPP regions only); and 0 if the dataset includes a minority of OHIBC regions.
    - Spatial resolution: Ideally, spatial data have sufficient resolution to distinguish between two neighboring OHIBC regions. Scored as 1.0 if the average spatial resolution is less than half the average area of OHIBC regions (e.g., census subdistricts; 4 km rasters of groundfish catch; Pacific Fishery Management Subareas); scored as 0.5 if the spatial resolution is on the order of the area of OHIBC regions (e.g, Pacific Fishery Management Areas; 0.5° rasters of species range and marine debris); and 0.0 if the data do not provide sufficient information to distinguish among regions (e.g., salmon stocks spatialized by river systems that do not indicate distribution in marine waters; province-level data on fisheries officers).
- Temporal dimension: OHIBC aims to identify changes in goal scores, pressures, and resilience annually across the study period 2001-2016.
    - Temporal extent: Ideally, temporal data span the entire study period, i.e. 2001-2016. Scored as 1.0 if the dataset spans 2001-2016; 0.5 if the dataset spans at least half of the study period; 0.0 if the dataset includes less than half the study period or is a static estimate of a time-varying indicator.
    - Temporal resolution: Ideally, temporal data would provide values at least annually. Scored as 1.0 if the dataset resolution is less than or equal to one year; 0.5 if the resolution is less than or equal to 10 years; and 0 if the dataset is static.
    - Temporal baseline: For those goal models that compare current condition to a historic reference point (note, not the same as trend). Scored 1.0 for data that allow comparisons to a benchmark at least 50 years prior; 0.5 for data that allow a benchmark at least 10 years prior.
- Fit dimension: OHIBC aims to capture a broad range of benefits afforded by a healthy marine social-ecological system, as well as the pressures and resilience that moderate those benefits. This dimension assesses how closely the available data "fit" the needs of the OHIBC target

calculation. This is rather subjective, as in some cases the available data were chosen to fit a goal model, while in others, a goal model required modification to accommodate the available data.

- Fit extent: Does the dataset adequately capture the full range of conceptual understanding required by the target calculation? Scored as 1.0 for data that inform understanding across the entire system (e.g., species condition information was available for nearly all the iconic species identified for the Iconic Species goal); scored 0.5 for data that may represent only a portion of benefits (e.g., species condition for the Species goal is limited to a subset of taxa assessed by IUCN and COSEWIC; the Salmon goal is based on a limited selection of indicator stocks). No datasets were scored 0.0.
- Fit resolution: Does the dataset allow for detailed exploration of goal status, pressures, or resilience within the broader context? Scored as 1.0 for datasets with a rich breakdown of categories or sectors (e.g., stock assessment and harvest data available for individual stocks; fishing license data can discriminate between First Nations and non-First Nations types); scored as 0.5 for datasets with some internal detail (e.g., census data include income and employment by very broad sectors in addition to overall); and 0.0 for data where finer-scale divisions are not available (e.g. aquaculture production potential is based on global averages but cannot be separated to identify potential for BC-specific species).

Layer-level scores

S13a Table: Data selection criteria by goal status layer

target	dataset	data score	spatial ext	spatial res	temporal baseline	temporal ext	temporal res	fit ext	fit res
Coastal Protection	exposure class	1.000	1.000	1.000				1.000	1.000
Coastal Protection	Land use change 1990-2010	0.714	1.000	1.000	0.500	0.500	0.500	0.500	1.000
Carbon Storage	Land use change 1990-2010	0.714	1.000	1.000	0.500	0.500	0.500	0.500	1.000
Wild-Capture Fisheries	B/Bmsy, F/Fmsy per spatialized stock	0.667	1.000	0.500		0.500	1.000	0.500	0.500
Wild-Capture Fisheries	Spatialized catch estimates	0.917	1.000	1.000		0.500	1.000	1.000	1.000
Aquaculture	harvest by management area	0.833	1.000	1.000		0.000	1.000	1.000	1.000
Aquaculture	Production potential per km2	0.750	1.000	1.000				1.000	0.000
Wild-capture Salmon	catch/exploitation estimates	0.417	0.000	0.000		0.500	1.000	0.500	0.500
Wild-capture Salmon	catch/exploitation targets	0.417	0.000	0.000		0.500	1.000	0.500	0.500
FN Resource Access Opps	commercial licenses	1.000	1.000	1.000		1.000	1.000	1.000	1.000
FN Resource Access Opps	escapement estimates	0.417	0.000	0.000		0.500	1.000	0.500	0.500
FN Resource Access Opps	escapement targets	0.417	0.000	0.000		0.500	1.000	0.500	0.500
FN Resource Access Opps	herring spawn index	1.000	1.000	1.000	1.000	1.000	1.000	1.000	1.000
FN Resource Access Opps	shellfish closures	0.667	1.000	1.000		0.000	1.000	0.500	0.500
Coastal Livelihoods	median income	0.833	1.000	1.000		1.000	0.500	1.000	0.500
Coastal Livelihoods	unemployment rate	0.833	1.000	1.000		1.000	0.500	1.000	0.500
Tourism & Recreation	Park visits	0.500	0.500	0.500		0.000	1.000	0.500	0.500
Tourism & Recreation	visitor center visits	0.583	0.500	0.500		0.500	1.000	0.500	0.500
Iconic Species	species condition	0.583	1.000	0.500		0.000	0.000	1.000	1.000
Iconic Species	species distribution	0.792	1.000	0.500				1.000	1.000
Iconic Species	species list	0.833	1.000					0.500	1.000
Lasting Special Places	tribal parks	0.444	0.000	1.000		0.000	0.000	1.000	1.000
Lasting Special Places	WDPA	1.000	1.000	1.000		1.000	1.000	1.000	1.000
Species	species condition	0.583	1.000	0.500		0.500	0.500	0.500	0.500
Species	species distribution	0.792	1.000	0.500				0.500	1.000
Habitats	EBSA locations	1.000	1.000	1.000				1.000	1.000
Habitats	salt marsh extent	0.714	1.000	1.000	0.500	0.500	0.500	0.500	1.000
Habitats	soft bottom extent	1.000	1.000	1.000				1.000	1.000
Habitats	trawl effort	0.833	1.000	1.000		0.500	1.000	1.000	0.500
Clean Waters	chemical pollution	0.667	1.000	1.000		0.500	0.500	0.500	0.500
Clean Waters	marine debris	0.333	0.500	0.500		0.000	0.000	0.500	0.500
Clean Waters	nutrient pollution	0.833	1.000	1.000		0.500	1.000	1.000	0.500
Clean Waters	pathogen pollution	0.833	1.000	1.000		0.500	0.500	1.000	1.000

S13bTable: Data selection criteria by pressure layer

target	dataset	data score	spatial ext	spatial res	temporal baseline	temporal ext	temporal res	fit ext	fit res
aq_benthic	sites above/below threshold for benthic inspections	0.500	1.000	0.000		0.000	1.000	0.500	0.500
aq_incidental	incidental fish take	0.667	1.000	0.000		0.000	1.000	1.000	1.000
aq_mammals	drowned mammals and predator control	0.667	1.000	0.000		0.000	1.000	1.000	1.000
cc_sst	SST pressure	0.857	1.000	1.000	0.500	0.500	1.000	1.000	1.000
cc_acid	OA pressure	0.917	0.500	1.000		1.000	1.000	1.000	1.000
cc_uv	UV pressure	0.643	1.000	0.000	0.000	0.500	1.000	1.000	1.000
cc_slr	SLR pressure	1.000	1.000	1.000		1.000	1.000	1.000	1.000
fp_fis_discards	Watson data	0.917	1.000	0.500		1.000	1.000	1.000	1.000
fp_fis_landings	Watson data	0.917	1.000	0.500		1.000	1.000	1.000	1.000
hd_intertidal	population density from census	0.667	1.000	1.000		1.000	0.500	0.000	0.500
hd_subtidal_sb	soft bottom extent	1.000	1.000	1.000				1.000	1.000
hd_logging	logging cutblock activity	0.833	1.000	1.000		1.000	1.000	0.500	0.500
po_chemical (eez and 3nmi)	chemical pollution	0.667	1.000	1.000		0.500	0.500	0.500	0.500
po_nutrient (eez and 3nmi)	nutrient pollution	0.833	1.000	1.000		0.500	1.000	1.000	0.500
po_pathogen	pathogen pollution	0.833	1.000	1.000		0.500	0.500	1.000	1.000
po_trash	marine debris	0.333	0.500	0.500		0.000	0.000	0.500	0.500
sp_alien	invasive risk by ecoregion	0.583	1.000	0.500		0.000	0.000	1.000	1.000
sp_genetic	genetic risk of non-native spp	0.625	1.000	0.000				0.500	1.000
sp_genetic	mariculture harvest	0.667	1.000	0.000		1.000	1.000	0.500	0.500
ss_cwbi (all, FN)	community well being index by csd	0.833	1.000	1.000		0.500	0.500	1.000	1.000

S13c Table: Data selection criteria by resilience layer

target	dataset	data score	spatial ext	spatial res	temporal baseline	temporal ext	temporal res	fit ext	fit res
cwbi (all, FN)	community well being index by csd	0.833	1.000	1.000		0.500	0.500	1.000	1.000
aq_regulation	aquaculture compliance	0.667	1.000	0.000		0.000	1.000	1.000	1.000
aq_regulation	aquaculture enforcement	0.667	1.000	0.000		0.000	1.000	1.000	1.000
fp_mpa (eez and 3nmi)	MaPP SMZs	0.875	0.500	1.000				1.000	1.000
fp_mpa (eez and 3nmi)	tribal parks	0.444	0.000	1.000		0.000	0.000	0.500	1.000
fp_mpa (eez and 3nmi)	WDPA	1.000	1.000	1.000		1.000	1.000	1.000	1.000
hd_mpa (eez and 3nmi)	MaPP SMZs	0.875	0.500	1.000				1.000	1.000
hd_mpa (eez and 3nmi)	tribal parks	0.444	0.000	1.000		0.000	0.000	0.500	1.000
hd_mpa (eez and 3nmi)	WDPA	1.000	1.000	1.000		1.000	1.000	1.000	1.000
fp_biomass_removal	fisheries act								
fp_biomass_removal	fisheries officers	0.667	1.000	0.000		1.000	1.000	0.500	0.500
fp_biomass_removal	licenses	0.800	1.000			1.000	1.000	0.500	0.500
fp_biomass_removal	observer coverage	0.667	1.000	0.000		1.000	1.000	0.500	0.500
hd_trawl_reduction	trawl reduction agreement								
mapp_resilience	MaPP process and plans	0.750	0.500	1.000		1.000	1.000	0.500	0.500
species_diversity (eez, 3nmi)	species condition	0.583	1.000	0.500		0.500	0.500	0.500	0.500
species_diversity (eez, 3nmi)	species distribution	0.792	1.000	0.500				0.500	1.000

Inclusion/exclusion of datasets based on these dimensions

Scoring datasets in this manner provides a useful heuristic to guide selection of datasets, by enabling comparison of the tradeoffs betwen two sets that may convey similar information. As an example, we can examine two datasets that were considered but not used in the OHIBC assessment. To inform our Wild-Capture Fisheries goal calculation, we used species-level catch data from DFO [6], available at fine resolution across the BC EEZ but spanning only a portion of the study period. We also considered data from the Sea Around Us Project [47] which provides catch reconstruction data at 0.5° spatial resolution, annually going back decades. Scoring the two data sets side by side, we see identical scores, trading spatial resolution for temporal resolution, at which point second-order criteria can come into play, in this case a preference for direct catch estimates over modeled catch reconstructions.

dataset	score	spatial res	spatial ext	temp. res	temp. ext	fit res	fit ext
DFO catch estimates	0.917	1.000	1.000	1.000	0.500	1.000	1.000
SAUP reconstructed catch	0.917	0.500	1.000	1.000	1.000	1.000	1.000

Similarly, we can compare aquaculture production datasets: we used DFO aquaculture production by Pacific Fishery Management Area [8], available for a short span of years, but also considered province-level estimates [48] spanning the entire study period. Again, the two datasets earn identical scores, trading spatial extent for temporal extent. Here we chose the spatially explicit data as more compatible with our production potential dataset [9].

dataset	score	spatial res	spatial ext	temp. res	temp. ext	fit res	fit ext
PFMA aquaculture harvest	0.833	1.000	1.000	1.000	0.000	1.000	1.000
Province aquaculture harvest	0.833	0.000	1.000	1.000	1.000	1.000	1.000

Note that this methodology as applied here implicitly places equal weights on each dimension, but preferentially weighting temporal qualities over spatial qualities (for example) could suggest different data selection decisions.

Goal-level scores

Goal-level scores are the average across all layers and facets used to calculate the goal (Table S14). As some layers provide more information than others (e.g. spatial-temporal vs. simply spatial), these layers contribute a greater weight to the goal score. For this reason, a goal's overall score may not be equal to the mean of its facet scores. In some cases, complementary datasets are included to improve extent (e.g. tribal parks to supplement parks and protected areas from WDPA). These complementary layers are combined prior to calculating the goal-level facet scores. The combined layer sums the full facet scores for the primary layer with half the facet scores for the secondary layer(s), with a maximum total value of 1.

S14a Table: Data selection criteria summarized to goal status

target	target score	fit ext	fit res	spatial ext	spatial res	temporal baseline	temporal ext	temporal res
Coastal Protection	0.818	0.750	1.000	1.000	1.000	0.500	0.500	0.500
Carbon Storage	0.714	0.500	1.000	1.000	1.000	0.500	0.500	0.500
Wild-Capture Fisheries	0.792	0.750	0.750	1.000	0.750		0.500	1.000
Aquaculture	0.800	1.000	0.500	1.000	1.000		0.000	1.000
Wild-capture Salmon	0.417	0.500	0.500	0.000	0.000		0.500	1.000
FN Resource Access Opps	0.710	0.700	0.700	0.600	0.600	1.000	0.600	1.000
Coastal Livelihoods	0.833	1.000	0.500	1.000	1.000		1.000	0.500
Iconic Species	0.731	0.833	1.000	1.000	0.500		0.000	0.000
Species	0.650	0.500	0.750	1.000	0.500		0.500	0.500
Habitats	0.857	0.875	0.875	1.000	1.000	0.500	0.500	0.750
Clean Waters	0.667	0.750	0.625	0.875	0.875		0.375	0.500

S14b Table: Data selection criteria summarized to pressure layer

target	target score	fit ext	fit res	spatial ext	spatial res	temporal baseline	temporal ext	temporal res
aq_benthic	0.500	0.500	0.500	1.000	0.000		0.000	1.000
aq_incidental	0.667	1.000	1.000	1.000	0.000		0.000	1.000
aq_mammals	0.667	1.000	1.000	1.000	0.000		0.000	1.000
cc_sst	0.857	1.000	1.000	1.000	1.000	0.500	0.500	1.000
cc_acid	0.917	1.000	1.000	0.500	1.000		1.000	1.000
cc_uv	0.643	1.000	1.000	1.000	0.000	0.000	0.500	1.000
cc_slr	1.000	1.000	1.000	1.000	1.000		1.000	1.000
fp_fis_discards	0.917	1.000	1.000	1.000	0.500		1.000	1.000
fp_fis_landings	0.917	1.000	1.000	1.000	0.500		1.000	1.000
hd_intertidal	0.667	0.000	0.500	1.000	1.000		1.000	0.500
hd_subtidal_sb	1.000	1.000	1.000	1.000	1.000			
hd_logging	0.833	0.500	0.500	1.000	1.000		1.000	1.000
po_chemical (eez and 3nmi)	0.667	0.500	0.500	1.000	1.000		0.500	0.500
po_nutrient (eez and 3nmi)	0.833	1.000	0.500	1.000	1.000		0.500	1.000
po_pathogen	0.833	1.000	1.000	1.000	1.000		0.500	0.500
po_trash	0.333	0.500	0.500	0.500	0.500		0.000	0.000
sp_alien	0.583	1.000	1.000	1.000	0.500		0.000	0.000
sp_genetic	0.650	0.500	0.750	1.000	0.000		1.000	1.000
ss_cwbi (all, FN)	0.833	1.000	1.000	1.000	1.000		0.500	0.500

S14c Table: Data selection criteria summarized to resilience layer

target	target score	fit ext	fit res	spatial ext	spatial res	temporal baseline	temporal ext	temporal res
cwbi (all, FN)	0.833	1.000	1.000	1.000	1.000		0.500	0.500
aq_regulation	0.667	1.000	1.000	1.000	0.000		0.000	1.000
fp_biomass_removal	0.706	0.500	0.500	1.000	0.000		1.000	1.000
mapp_resilience	0.750	0.500	0.500	0.500	1.000		1.000	1.000
species_diversity (eez, 3nmi)	0.650	0.500	0.750	1.000	0.500		0.500	0.500

References for Supporting Information

1. Agriculture and Agri-Food Canada. Land Use 1990, 2000 & 2010 - Open Government Portal. https://open.canada.ca/data/en/dataset/18e3ef1a-497c-40c6-8326-aac1a34a0dec; 2015.

2. British Columbia Marine Conservation Analysis Project Team. Marine Atlas of Pacific Canada: A Product of the British Columbia Marine Conservation Analysis. BC Marine Conservation Analysis. www.bcmca.ca. 2011.

3. Sharp R, Chaplin-Kramer R, Wood S, Guerry A, Tallis H, Ricketts T. Integrated Valuation of Ecosystem Services and Tradeoffs. 2018; 307.

4. Mcleod E, Chmura GL, Bouillon S, Salm R, Björk M, Duarte CM, et al. A blueprint for blue carbon: Toward an improved understanding of the role of vegetated coastal habitats in sequestering CO2. Frontiers in Ecology and the Environment. 2011;9: 552–560.

5. Re3data.Org. RAM Legacy Stock Assessment Database. 2016. doi:10.17616/r34d2x

6. Fisheries and Oceans Canada. Fisheries catch estimates. Government of Canada; 2018.

7. Free C. RAM Legacy Stock Boundary Database | Christopher M. Free. 2017.

8. Fisheries and Oceans Canada. Aquaculture production PFMA 2011-15. Government of Canada; 2017.

9. Gentry RR, Lester SE, Kappel CV, White C, Bell TW, Stevens J, et al. Offshore aquaculture: Spatial planning principles for sustainable development. Ecology and Evolution. 2017;7: 733–743. doi:10.1002/ece3.2637

10. Fisheries and Oceans Canada. Aquaculture tenures. Government of Canada; 2017.

11. LGL Limited, Pacific Salmon Foundation. North and Central Coast Salmon Run Reconstruction. http://shiny.lglsidney.com/ncc-salmon/; 2017.

12. Pacific Salmon Commission. Pacific Salmon Commission Annual Reports. 2017.

13. Fisheries and Oceans Canada. Salmon exploitation and escapement rates and targets 2005-2015. Government of Canada; 2017.

14. Fisheries and Oceans Canada. Contaminated Fisheries Prohibition Orders 2009-2015. Government of Canada; 2017.

15. Fisheries and Oceans Canada. DFO Pacific Region Commercial Licences. Government of Canada; 2017.

16. Fisheries and Oceans Canada. Herring section spawn tables (1940-2016) Tableaux de Pontes de sections de hareng. http://www.pac.dfo-mpo.gc.ca/science/species-especes/pelagic-pelagique/herring-hareng/herspawn/tabsbkm2-eng.html; 2016.

17. Statistics Canada. Median household income and Unemployment rate by census subdistrict, 1996, 2001, 2006, 2011, 2016. 2017.

18. Province of British Columbia: BC Parks Parks. BC Parks End of Year Reports. http://www.env.gov.bc.ca/bcparks/research/; 2017.

19. Province of British Columbia: Destination BC. HelloBC Visitor Centres Listing - Data Catalogue. https://catalogue.data.gov.bc.ca/dataset/2e264b91-5936-4e08-a79f-6236a20feeda; 2016.

20. IUCN. The IUCN Red List of Threatened Species. Version 2018-1. http://www.iucnredlist.org; 2018.

21. BirdLife International and Handbook of the Birds of the World. Bird species distribution maps of the world. Version 7.0. BirdLife International, Cambridge, UK and NatureServe, Arlington, USA; 2018.

22. Kaschner K, Rius-Barile J, Kesner-Reyes K, Garilao C, Kullander S, Rees T, et al. AquaMaps: Predicted range maps for aquatic species. www.aquamaps.org; 2016.

23. BC Ministry of Environment. BC Species & Ecosystems Explorer - Province of British Columbia. https://www2.gov.bc.ca/gov/content/environment/plants-animals-ecosystems/conservation-data-centre/explore-cdc-data/species-and-ecosystems-explorer; 2018.

24. IUCN, UNEP-WCMC. The World Database on Protected Areas (WDPA). Cambridge, UK: UNEP-WCMC. www.protectedplanet.net; 2018.

25. GeoBC - Ministry of Forests, Lands, Natural Resource Operations and Rural Development. Freshwater Atlas Watersheds - Data Catalogue. https://catalogue.data.gov.bc.ca/dataset/freshwater-atlas-watersheds; 2011.

26. O'Hara C. OHIBC: An Ocean Health Index Assessment for British Columbia. GitHub; 2019.

27. Fisheries and Oceans Canada. Ecologically or Biologically Significant Marine Areas. Government of Canada; 2016.

28. Halpern BS, Frazier M, Afflerbach J, Lowndes JS, Micheli F, O'Hara C, et al. Recent pace of change in human impact on the world's ocean. Scientific Reports. 2019;9: 1–8. doi:10.1038/s41598-019-47201-9

29. Indigenous and Northern Affairs Canada. National Assessment of First Nations Water and Wastewater Systems - National Roll-Up Report. 2011 Aug.

30. Environment Canada. 2011 Municipal Water Use Report Municipal Water Use 2009 Statistics. http://www.ec.gc.ca/doc/publications/eau-water/COM1454/index-eng.htm; 2011.

31. Environment Canada. 2010 Municipal Water Use Report Municipal Water Use, 2006 Statistics. 2010.

32. Environment Canada. 2007 Municipal Water Use Report Municipal Water Use, 2004 Statistics. 2007.

33. Van Sebille E, Wilcox C, Lebreton L, Maximenko N, Hardesty BD, Van Franeker JA, et al. A global inventory of small floating plastic debris. Environmental Research Letters. 2015;10: 124006.

34. Fisheries and Oceans Canada. DFO marine finfish aquaculture audit activities in BC. http://www.pac.dfo-mpo.gc.ca/aquaculture/reporting-rapports/mer-mar-audit-verif/index-eng.html; 2018.

35. Watson RA. A database of global marine commercial, small-scale, illegal and unreported fisheries catch 19502014. Scientific Data. 2017;4. doi:10.1038/sdata.2017.39

36. Canada Ministry of Forests, Lands, Natural Resource Operations and Rural Development. Harvested Areas of BC (Consolidated Cutblocks) - Data Catalogue. https://catalogue.data.gov.bc.ca/dataset/harvested-areas-of-bc-consolidated-cutblocks-; 2017.

37. Molnar JL, Gamboa RL, Revenga C, Spalding MD. Assessing the global threat of invasive species to marine biodiversity. Frontiers in Ecology and the Environment. 2008;6: 485–492. doi:10.1890/070064

38. Trujillo P. Using a mariculture sustainability index to rank countries' performances. A comparative assessment of biodiversity, fisheries and aquaculture in. 2008;53: 28–56.

39. Aboriginal Affairs and Northern Development Canada. The Community Well-Being Index: Report on Trends in First Nations Communities, 1981-2011. Ottawa: Her Majesty the Queen in Right of Canada, represented by the Minister of Aboriginal Affairs and Northern Development; 2015.

40. Fisheries and Oceans Canada. Aquaculture Regulations and Compliance, Pacific Region. http://www.pac.dfo-mpo.gc.ca/aquaculture/regs-eng.html; 2010.

41. Government of Canada National Research Council Canada. Pacific Region integrated fisheries management plan, groundfish, effective February 21, 2016. http://science-catalogue.canada.ca/record=4017949~S6; 2016.

42. Environment and Climate Change Canada. Canadian protected areas status report 2012-2015. Government of Canada; 2016.

43. Wallace S, Turris B, Driscoll J, Bodtker K, Mose B, Munro G. Canada's Pacific groundfish trawl habitat agreement: A global first in an ecosystem approach to bottom trawl impacts. Marine Policy. 2015;60: 240–248. doi:10.1016/j.marpol.2015.06.028

44. Hughes TP. Climate Change, Human Impacts, and the Resilience of Coral Reefs. Science. 2003;301: 929–933. doi:10.1126/science.1085046

45. Halpern BS, Longo C, Lowndes JSS, Best BD, Frazier M, Katona SK, et al. Patterns and Emerging Trends in Global Ocean Health. Tsikliras AC, editor. PLOS ONE. 2015;10: e0117863. doi:10.1371/journal.pone.0117863

46. Fritz S, See L, Carlson T, Haklay M(, Oliver JL, Fraisl D, et al. Citizen science and the United Nations Sustainable Development Goals. Nature Sustainability. 2019;2: 922–930. doi:10.1038/s41893-019-0390-3

47. Pauly D, Zeller D, editors. Sea Around Us Concepts, Design and Data. seaaroundus.org; 2015.

48. Government of Canada F and OSS. Production Quantities and Values | Fisheries and Oceans Canada. http://www.dfo-mpo.gc.ca/stats/aqua/aqua-prod-eng.htm; 2016.
