# Supplementary material for: Changes in ocean health in British Columbia from 2001 to 2016
Source: PLoS One. 2020 Jan 30;15(1):e0227502. doi: 10.1371/journal.pone.0227502 (PMC6992189; doi:10.1371/journal.pone.0227502)
Supplement: Attachment — Submitted filename: response_to_reviewers.pdf [file pone.0227502.s002.pdf]

Casey O'Hara
2400 Bren Hall, UC Santa Barbara
Santa Barbara CA 93106
cohara@bren.ucsb.edu

November 12, 2019

PLOS ONE

Dear Dr. Belgrano,

Thank you for the thoughtful feedback on our manuscript, "Changes in ocean health in British Columbia from 2001 to 2016." We have addressed all the reviewer's concerns and suggestions, and feel that the resulting manuscript is now much stronger. Below, we outline the changes we have made in response to the reviewer's suggestion.

Reviewer #1: I really enjoyed reading this interesting piece of work. I found it appropriate and necessary, specially to address the regional scale of the OHI.

However, I think that the paper would benefit from explaining in more detail the incomplete datasets. For example, in lines 149-151 authors say "In such cases, datasets were chosen based on careful consideration of the tradeoffs among spatial resolution, temporal resolution, and how well the data represented the needs of the assessment." Could you provide a specific example of how you did it?

We have now included details on this process in the Supporting Information Methods, in its own section titled "Data selection criteria" starting on line 559. We rank spatial resolution and extent, temporal resolution and extent (and baseline where appropriate), and thematic "fit" and "resolution" of each dataset to its particular status, pressure, or resilience calculation. Each dimension is scored on a 0.0/0.5/1.0 scale based on criteria described in the supporting methods. In S13 Table, the relevant scores are averaged for each dataset as a heuristic for selection and comparison. As an example of using this method as data selection criteria, we compare two datasets included in the OHIBC assessment against two that we considered, but decided against.

In S14 Table, we aggregate dataset scores to calculate scores for each goal status, pressure, and resilience layers.

Within the manuscript itself, we have included notes referring to these SI Methods in several locations:

- Lines 150-153: "In such cases, datasets were chosen based on careful consideration of the tradeoffs among spatial extent and resolution, temporal extent and resolution, and how well the data represented the needs of the assessment (see SI methods, S13 and S14 Tables)."
- Lines 610-612: "Giving explicit voice to ideals of ocean health, and to reference points against which these ideals can be measured, highlights in many cases the gaps and lack of critically important data (see SI methods, S13 and S14 Tables)."
- Lines 673-675: "…we encountered challenges in accessing environmental, social, and economic data at spatial, sectoral, and temporal resolutions relevant to conservation policy makers (see SI Methods, S13 and S14 Tables)."

Note also changes in lines 248-252 to include both spatial and temporal *extent* and *resolution* (original text just mentioned *resolution*.)

Again, in lines 169-172 the authors state that "To address incomplete data sets, we applied two gapfilling procedures. For periodic data, we estimated intervening years using a linear interpolation between available

data years. For truncated data, we typically expanded the time series using last observation carried forward and/or first observation carried back methods". How you did it? By using expert consultation? Did other stakeholders validated the results? How?

We chose linear interpolation and Last Observation Carried Forward/Next Observation Carried Back (LOCF/NOCB) as simple, transparent gapfilling procedures that are commonly used including in global OHI assessments. To make our methods more transparent, we have explicitly noted in the SI Methods where linear interpolation and/or LOCF/NOCB extrapolation have been used for each goal. In the line-numbered version of SI (attached for convenience), see lines 83, 103, 141, 161, 172, 195, 204, 209, 221, 226, 267, 281, 295, 325, 349, 368, 375, 388, 397. The text of these additions is omitted here for brevity. Note also minor changes to the manuscript text in lines 272-275: "To address incomplete data sets, we applied two gapfilling procedures *chosen for simplicity and transparency*. For periodic data, we estimated intervening years using a linear interpolation between available data years. For truncated data, we typically expanded the time series using last observation carried forward and/or *next* observation carried back *extrapolation* methods."

Lines 525-528: the lack of cultural availability of data is an important gap, but at the same time, an excellent window of opportunity to collect data through, e.g., in-depth interviews or deliberative mapping.

This is an excellent point - we have added a reference in line 626 to highlight this opportunity: "…may indicate an opportunity to refine goal models, redefine reference points, conduct assessments at even finer scales, or generate relevant data and insights through methods such as in-depth interviews or deliberative mapping."

Management: did the authors discuss the process of the OHI with regional/local representatives of the administration? Would be really strong to include this in more detail into the discussion, because it will help policy makers to encourage them to use the OHI.

This is an excellent suggestion, but we do not wish to overstate the role of administrators in the process. In the initial phases of this assessment, we discussed the OHI process with representatives of First Nations and the Province of British Columbia via MaPP, engaging them to help define the goals and align them with local and regional needs and priorities. Beyond this planning phase, our engagement with administration has primarily been in a scientific advisory role through DFO scientists (including coauthors Karen Hunter and R. Ian Perry) rather than policymakers. Because our funding source is also involved in MaPP, we wished to maintain a degree of independence between the planning efforts of MaPP and the resulting calculations of OHIBC, to avoid the appearance of a conflict of interest.

Thank you for your time and effort in reviewing our manuscript. We believe our revisions have addressed all comments thoroughly.

Sincerely, on behalf of all coauthors,

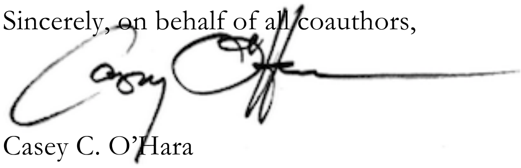

Casey C. O'Hara
Bren School of Environmental Science & Management
University of California, Santa Barbara
cohara@bren.ucsb.edu
